# Phylogeny and adaptive evolution of subgenus *Rhizirideum* (Amaryllidaceae, *Allium*) based on plastid genomes

**DOI:** 10.1186/s12870-022-03993-z

**Published:** 2023-02-01

**Authors:** Xiao Fu, Deng-Feng Xie, Yu-Yang Zhou, Rui-Yu Cheng, Xiang-Yi Zhang, Song-dong Zhou, Xing-Jin He

**Affiliations:** Key Laboratory of Bio-Resources and Eco-Environment of Ministry of Education, College of Life Sciences, Sichuan University, 610065 Chengdu, Sichuan The People’s Republic of China

**Keywords:** *Allium*, Subgenus *Rhizirideum*, Plastid genomes, Adaptive evolution, Phylogeny

## Abstract

The subgenus *Rhizirideum* in the genus *Allium* consists of 38 species worldwide and forms five sections (*A*. sect. *Rhizomatosa, A*. sect. *Tenuissima, A*. sect. *Rhizirideum, A*. sect. *Eduardia,* and *A*. sect. *Caespitosoprason*), *A*. sect. *Caespitosoprason* being merged into *A*. sect. *Rhizomatosa* recently. Previous studies on this subgenus mainly focused on separate sections. To investigate the inter-section and inter-subgenera phylogenetic relationships and adaptive evolution of *A*. subg. *Rhizirideum*, we selected thirteen representative species, which cover five sections of this subgenus and can represent four typical phenotypes of it. We conducted the comparative plastome analysis with our thirteen plastomes. And phylogenetic inferences with CDSs and complete sequences of plastomes of our thirteen species and another fifty-four related species were also performed. As a result, the *A*. subg. *Rhizirideum* plastomes were relatively conservative in structure, IR/SC borders, codon usage, and repeat sequence. In phylogenetic results, the inter-subgenera relationships among *A*. subg. *Rhizirideum* and other genus *Allium* subgenera were generally similar to the previous reports. In contrast, the inter-section relationships within our subgenus *A*. subg. *Rhizirideum* were newly resolved in this study. *A*. sect. *Rhizomatosa* and *A*. sect. *Tenuissima* were sister branches, which were then clustered with *A*. sect. *Rhizirideum* and *A*. sect. *Eduardia* successively. However, *Allium Polyrhizum* Turcz. ex Regel*,* type species of *A*. sect. *Caespitosoprason*, was resolved as the basal taxon of *A*. subg. *Rhizirideum*. *Allium siphonanthum* J. M. Xu was also found in clade *A*. subg. *Cyathophora* instead of clade *A*. subg. *Rhizirideum*. The selective pressure analysis was also conducted, and most protein-coding genes were under purifying selection. At the same time, just one gene, *ycf2*, was found under positive selection, and another three genes (*rbcL*, *ycf1a*, *ycf1b*) presented relaxed selection, which were all involved in the photosynthesis. The low temperature, dry climate, and high altitude of the extreme habitats where *A*. subg. *Rhizirideum* species grow might impose intense natural selection forces on their plastome genes for photosynthesis. In summary, our research provides new insights into the phylogeny and adaptive evolution of *A*. subg. *Rhizirideum*. Moreover, we suggest that the positions of the A. subg. *Rhizirideum* species *A. polyrhizum* and *A. siphonanthum* should be reconsidered.

**Supplementary Information:**

The online version contains supplementary material available at 10.1186/s12870-022-03993-z.

## Introduction

*Allium* (Allioideae, Amaryllidaceae), one of the largest genera of monocots, has more than 900 verified species on the Earth [1]. Many species in this genus have been used for edible (e.g., *A. sativum*, *A. tuberosum*, *A. porrum*), medicinal (e.g., *A. sativum*, *A. victorialis*, *A. cepa*), and ornamental (e.g., *A. giganteum*, *A. wallichii*, *A. moly*). Genus *Allium* was originally established by Linnaeus in *Species plantarum* [2], which initially contained only 30 *Allium* species sorted into three alliances. Subsequently, many scientists published a large quantity of new *Allium* taxa, and analyses on the taxonomy and phylogeny of *Allium* also emerged because of the complicated relationship within this genus. Regel’s monograph included 263 species and distributed them into six sections [3, 4]. Afterwards, Traub [5] sorted 600 *Allium* species into three subgenera, including 36 sections and subsections. Wendelbo [6] first proposed the subgenus *Rhizirideum*. After that, Kamelin [7] revised the phylogeny of *Allium* and classified it into six subgenera (44 sections & subsections). In Kamelin’s taxonomy, the subgenus *Rhizirideum* contained 150 species, such as *A. cepa*, *A. senescens*, and *A. ramosum* and further sorted into 12 sections and subsections as Sect. *cepa*, Sect. *Butomissa*, Sect. *Rhizirideum*. Later, Friesen et al. [8] reconstructed the phylogeny of *Allium* based on ITS data and divided it into three main evolutionary lineages. Friesen et al. [8] put the new subgenus *Rhizirideum* forward (*A*. subg. *Rhizirideum* in the following) and distributed approximately 780 *Allium* species into 15 subgenera (72 sections)*.* At the same time, the previous subgenus *Rhizirideum* was disproved and found to be nonmonophyletic.

Sixteen species (e.g., *Allium senescens* L*.*) in the previous subgenus *Rhizirideum* were still assorted into the new one, while the others were assorted into other subgenera such as *Anguinum* (e.g., *Allium Victorialis* L.), *Cepa* (e.g., *Allium Cepa* L*.*), and *Butomiss*a (e.g., *Allium Ramosum* L*.*). At that time, subgenus *Rhizirideum* included five sections: *Rhizirideum*, *Rhizomatosa*, *Tenuissima*, *Eduardii*, and *Caespitosoprason*. Recently, [9] provided adequate evidence for the monophyly of subgenus *Rhizirideum* based on chloroplast DNA fragments data. Friesen et al. [10] merged section *Caespitosoprason* into section *Rhizomatosa* under subgenus *Rhizirideum.*

**Fig. 1:**
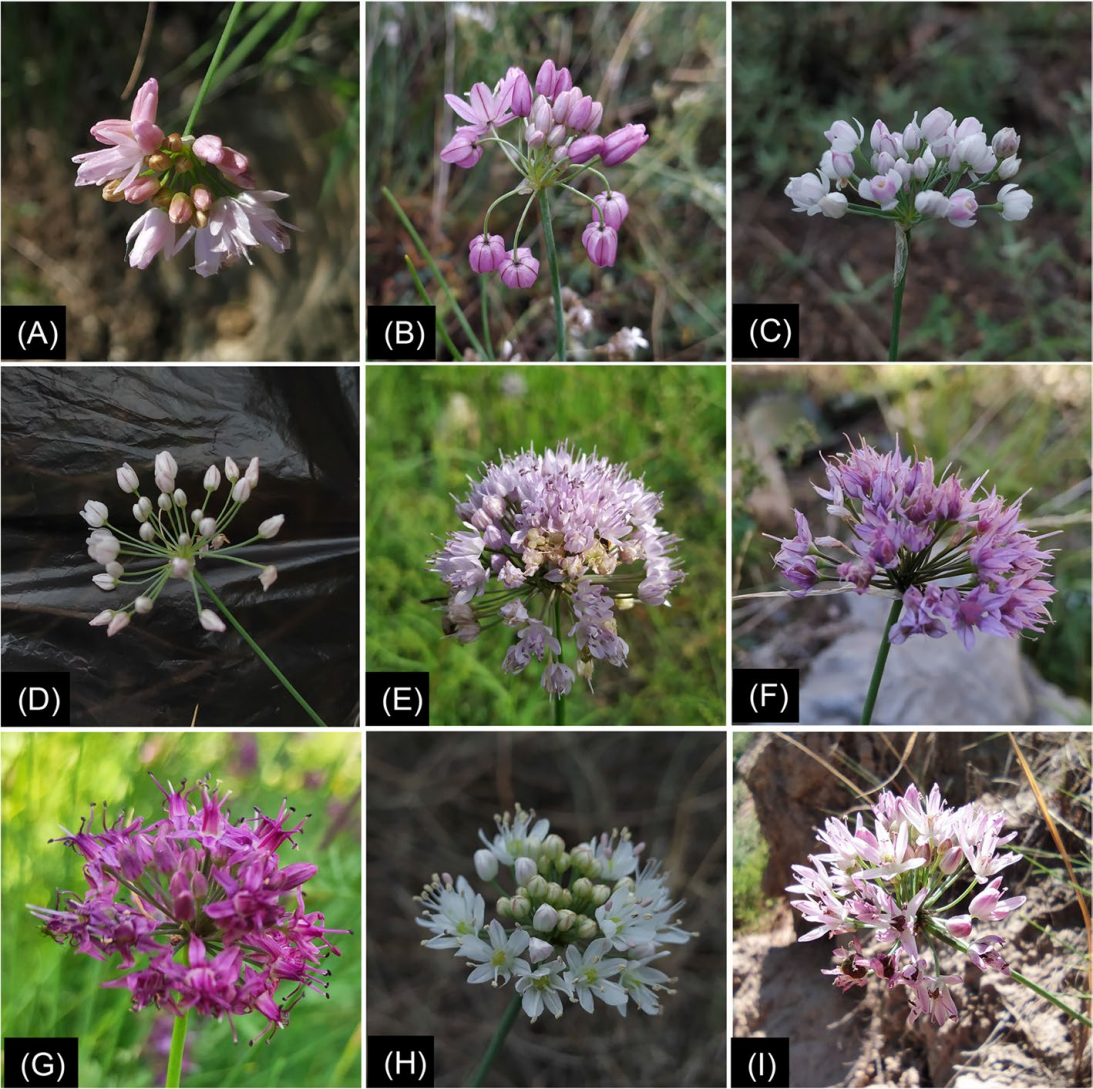
Inflorescences of eight species in A. subg. *Rhizirideum*. (**A**), *A. bidentatum*; (**B**), *A. mongolicum*; (**C**), *A. anisopodium*; (**D**),* A. tenuissimum*; (**E**), *A. senescens*; (**F**), *A. eduardii*; (**G**), *A. przewalskianum*; (**H**) & (**I**), *A. polyrhizum*

Currently, subgenus *Rhizirideum* consists of four sections (*Rhizirideum*, *Rhizomatosa*, *Tenuissima*, *Eduardii*) and thirty-eight species in total [8, 11] (Additional file 2: Table S1). It was located in the third lineage of the *Allium* phylogeny. Species in this subgenus were characterised by obvious rhizome, leaves subcylindrical to flat, perianth white to purple, ovary with two ovules per locule, and inner filaments broadened at the base (Fig. 1, Additional file 1: Fig. S1).

Studies of species in subgenus *Rhizirideum* have been conducted frequently in the past century [8, 10, 12–23]. It was found that the chromosome base number of this subgenus was eight, and the ploidy was mainly 2x or 4x. And Species in this subgenus spread over the Eurasian steppe. Sinitsyna et al. [24] divided section *Rhizirideum* into two geographical groups, the Asiatic and European groups. The diversification and speciation of this section coincide with the history of the modern Eurasian steppe. Meanwhile, the latest study of section *Rhizomatosa* on biogeography indicated that species in this section were distributed in the Central Asian steppe, and the distribution was in accordance with the history of the landscape and climate [10]. However, some phylogenetic studies were focused on the previous subgenus *Rhizirideum,* and the others focused on section *Rhizirideum* and section *Rhizomatosa*. Phylogenetic analysis on section *Tenuissima* and section *Eduardii* were lacking, so more fieldwork and further investigation should be undertaken.

In recent years, the complete chloroplast genome has been popular for its conservative structure, low recombination rate, and enormous genetic information. It has been widely used in the phylogenetic reconstruction and adaptive evolution [25–27]. Several *Allium* taxa have also been studied on their plastomes, for instance, section C*epa*, *Daghestanica*, and subgenus *Cyathophora* [28–30]*.* Xie et al. [31] reconstructed the phylogenetic relationship of the genus *Allium* with thirty-nine complete chloroplast genomes and revealed the evolutionary features of *Allium*. However, similar studies on the subgenus *Rhizirideum* have not yet been reported. Previous phylogenetic studies of subgenus *Rhizirideum* were primarily based on ITS or plastid DNA fragments, which provided limited information for infrageneric relationships. Furthermore, the analysis of adaptive evolution was also inadequate*.* Thus it is necessary to investigate further the composition, structure, and evolution of subgenus *Rhizirideum* plastomes. We collected thirteen species in Subgenus *Rhizirideum* and combined fifty-four related species to conduct comparative chloroplast genome analyses. Our aims are as follows: (1) to compare the structures and genetic compositions of plastomes of thirteen *Rhizirideum* species and (2) to reconstruct the phylogeny of subgenus *Rhizirideum* as well as some related *Allium* subgenera; (3) to analyse the adaptive evolution of subgenus *Rhizirideum* species.

## Results

### Plastome structure of subgenus *Rhizirideum* species

**Fig. 2:**
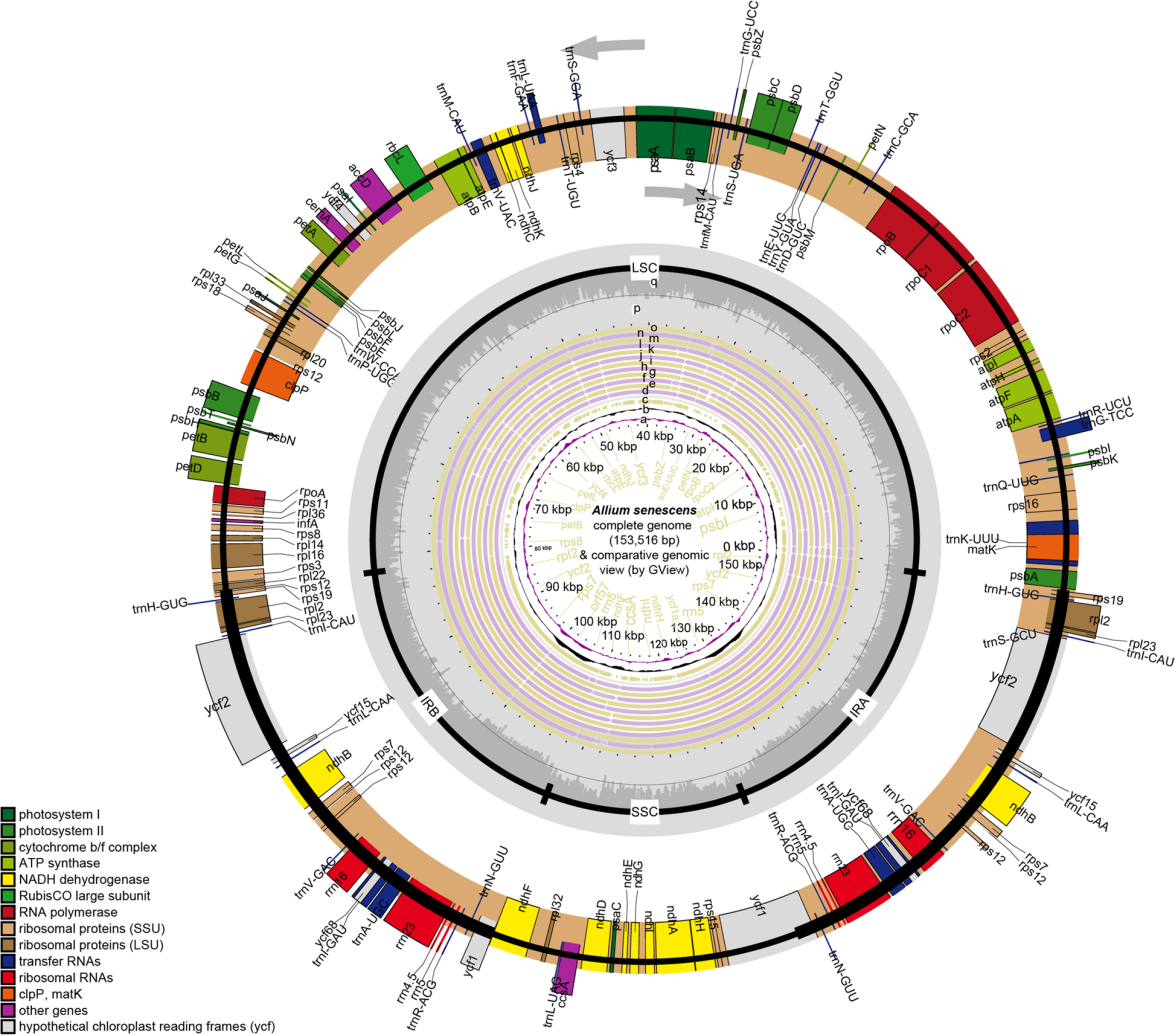
Chloroplast map of *Allium senescens* (the outermost circle and rings p-q) and GView comparison of thireteen A. subg. Rhizirideum plastomes (rings c-o). Genes are shown in different colors based on their functional groups. Genes on the inside of the outermost circle are transcribed clockwise, and those on the outside counter-clockwise. LSC, large single-copy region; SSC, small single-copy region; IR, inverted repeat. Ring a: GC content deviation from the average GC content of *A. senescens*, waves inside lower than the average, waves outside higher than the average. Ring b: GC skew of *A. senescens*, GC skew = (G-C)/(G + C), waves inside > 0, waves outside < 0. Ring c: reference of multiple alignments (*A. senescens* plastome). Rings d-n denote the result of multiple alignments of plastomes outwards in turn: *A. polyrhizum*, *A. bidentatum*, *A. dentigerum*, *A. caespitosum*, *A. mongolicum*, *A. anisopodium*, *A. tenuissimum*, *A. spirale*, *A. nutans*, *A. eduardii*, *A. przewalskianum*, *A. siphonanthum*. Ring p: GC content of *A. senescens*. Ring q: AT content of *A. senescens*

The subgenus *Rhizirideum* plastomes shared a quadripartite circular structure with two inverted repeats (IRa & IRb), one large single copy (LSC), and one small single copy (SSC) (Fig. 2, Table 1). The sizes of thirteen *Rhizirideum* plastomes ranged from 153,723 bp to 153,257 bp, and their overall GC content ranged from 36.8 to 36.9%. Each plastome contained 141 genes, among which 87 or 89 protein-coding sequences, 38 tRNA-coding genes, and 8rRNA-coding genes. Moreover, 26 genes were interpreted by introns (Table 2). The genes *clpP*, *rps12*, and *ycf3* had two introns inserted into their sequences. Moreover, the *trnK-UUU* gene had the longest intron, where the *matK* gene was located. The *rps12* is a trans-spliced gene with the 5′-end in the LSC region and the duplicated 3′-ends in the IR regions.

**Table 1:** Summary of the subgenus *Rhizirideum* plastomes

Taxon	Total genome length (bp)	GC (%)	IR length (bp)	LSC length (bp)	SSC length (bp)	Gene	CDS	tRNA	rRNA
*A. polyrhizum*	153,614	36.8	26,450	82,624	18,090	141	89 (9)	38 (8)	8 (4)
*A. bidentatum*	153,443	36.8	26,459	82,504	18,021	141	89 (9)	38 (8)	8 (4)
*A. dentigerum*	153,538	36.8	26,625	82,522	17,766	141	89 (9)	38 (8)	8 (4)
*A. caespitosum*	153,667	36.8	26,490	82,643	18,044	141	87 (9)	38 (8)	8 (4)
*A. mongolicum*	153,667	36.8	26,490	82,645	18,042	141	87 (9)	38 (8)	8 (4)
*A. anisopodium*	153,407	36.8	26,491	82,426	17,999	141	87 (9)	38 (8)	8 (4)
*A. tenuissimum*	153,459	36.8	26,491	82,484	17,993	141	87 (9)	38 (8)	8 (4)
*A. senescens*	153,516	36.8	26,491	82,548	17,986	141	89 (9)	38 (8)	8 (4)
*A. spirale*	153,549	36.8	26,493	82,576	17,987	141	89 (9)	38 (8)	8 (4)
*A. nutans*	153,456	36.9	26,487	82,531	17,951	141	87 (9)	38 (8)	8 (4)
*A. eduardii*	153,497	36.9	26,732	82,296	17,737	141	89 (9)	38 (8)	8 (4)
*A. przewalskianum*	153,257	36.9	26,437	82,410	17,973	141	89 (9)	38 (8)	8 (4)
*A. siphonanthum*	153,723	37.9	26,495	82,752	17,981	141	89 (9)	38 (8)	8 (4)

Numbers in brackets note the number of double-copy genes

**Table 2:** Summary of genes interrupted by introns in *Rhizirideum* plastomes

No.	Gene	Region	Exon I (bp)	Intron I (bp)	Exon II (bp)	Intron II (bp)	Exon III (bp)
1	*atpF*	LSC	144^+^	789	411^+^		
2	*clpP*	LSC	69^+^	1094	294^+^	879	252^+^
3	*ndhA*	SSC	558^+^	1128	540^+^		
4	*ndhB*	IRb	777^+^	701	756^+^		
5	*ndhB*	IRa	777^−^	701	756^−^		
6	*petB*	LSC	6^−^	920	642^−^		
7	*petD*	LSC	8^−^	746	514^−^		
8	*rpl16*	LSC	9^+^	1042	399^+^		
9	*rpl2*	IRb	387^+^	659	432^+^		
10	*rpl2*	IRa	387^−^	659	432^−^		
11	*rpoC1*	LSC	432^+^	759	1623^+^		
12	*rps12a*	LSC, IRa	114^+^	69,984	232^−^	542	26^−^
13	*rps12b*	LSC,IRb	114^+^	28,994	232^+^	542	26^+^
14	*rps16*	LSC	40^+^	846	197^+^		
15	*trnA-UGC*	IRb	38^−^	815	35^+^		
16	*trnA-UGC*	IRa	38^+^	815	35^+^		
17	*trnG-TCC*	LSC	23^−^	692	49^−^		
18	*trnI-GAU*	IRb	37^−^	934	35^−^		
19	*trnI-GAU*	IRa	37^+^	934	35^+^		
20	*trnL-UAA*	LSC	35^−^	305	50^−^		
21	*trnV-UAC*	LSC	37^+^	598	37^+^		
22	*ycf3*	LSC	129^+^	722	228^+^	738	
23	*ycf68*	IRb	42^−^	31	411^−^		153^+^
24	*ycf68*	IRa	42^+^	31	411^+^		
25	*trnK-UUU*	LSC	37^+^	2564	35^+^		

The data in this table is from *Allium senescens* plastome

**Fig. 3:**
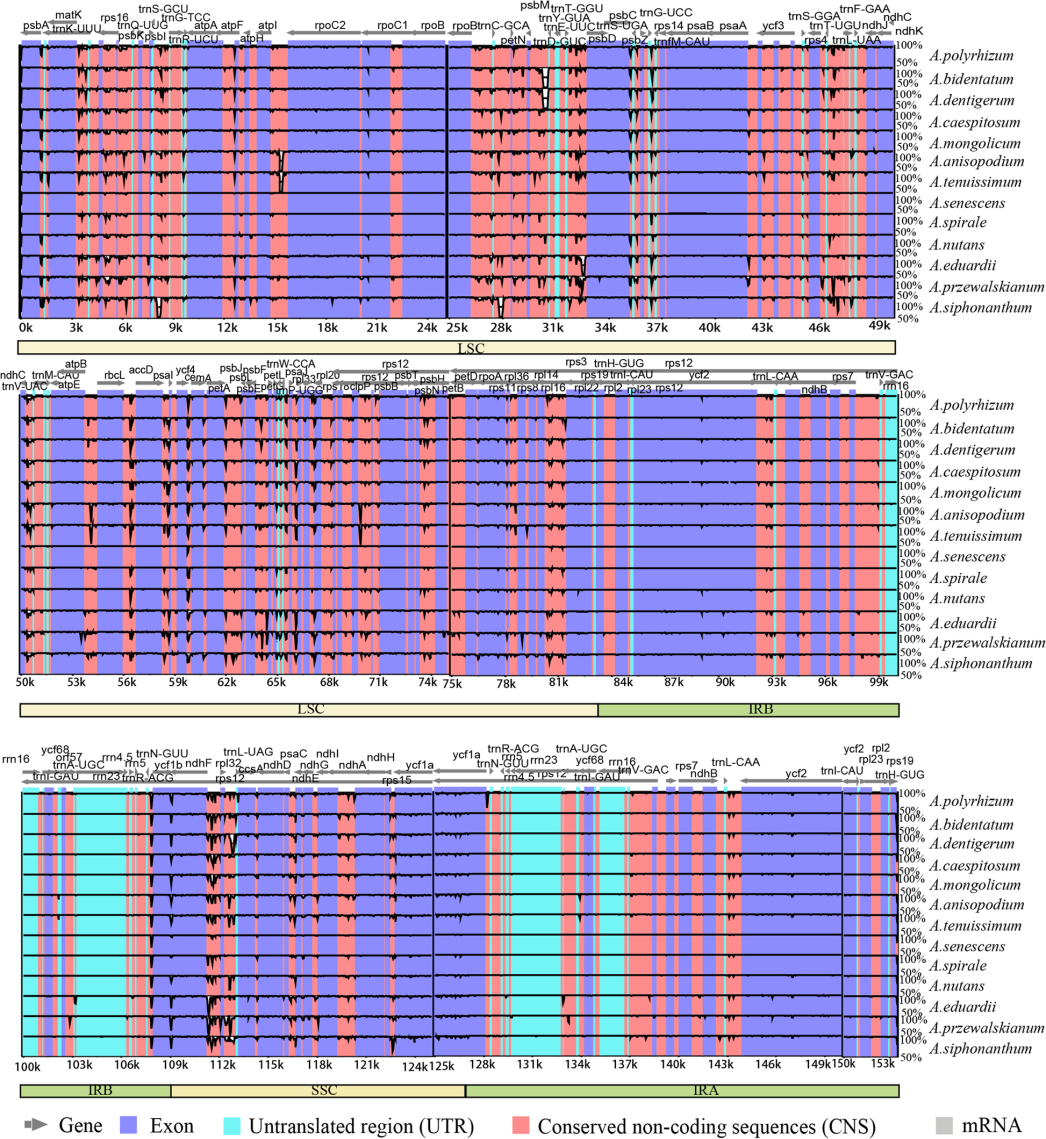
mVISTA comparison of thirteen *A*. subg. *Rhizirideum* plastomes (*A. senescens* as reference).

Multiple alignments of *Rhizirideum* plastomes showed similar structural features (Fig. 2). Matching distribution patterns of GC islands were displayed among thirteen *Rhizirideum* plastomes (Fig. 2, rings a-b). IR regions showed a GC skew < 0 (G < C) while most areas of LSC and SSC regions showed a GC skew > 0 (G > C) (Fig. 2, ring b). LSC and SSC regions, especially LSC, showed lower sequence identities than IR regions (Fig. 2, rings c-o). There was only one common gap in each IR region, *ycf2-trnI* CAU and *rrn16-trnI* GAU, respectively (Fig. 2). However, in the single copy (SC) regions, *Rhizirideum* species shared several divergent sequence sites (Fig. 2): (1) *psbA-rps19*, (2) *matK-trnK* UUU (10 species except for *Allium bidentatum* Fisch. ex Prokh. et Ikonnikov-Galitzky*, Allium dentigerum* Prokh*.* and *Allium spirale* Willdenow), (3) *psbD-trnT* GGU (10 species except for *Allium mongolicum* Regel*, Allium anisopodium* Ledeb. and *A. spirale*), (4) *trnG UCC* – *trnfM CAU* (12 species except for *A.spirale*), (5) *ndhC-ndhK*, (6) *petA-psbJ* (11 species except for *A. spirale* and *Allium nutans L.*). Besides, the diagram drawn by mVISTA (Fig. 3) showed sequence identities of different regions in *Rhizirideum* plastomes straightforward regarding *A. senescens* as a reference. As it indicated, exon regions had higher identity values than UTR and CNS regions. IR regions also had higher sequence identities than SC regions.

**Fig. 4:**
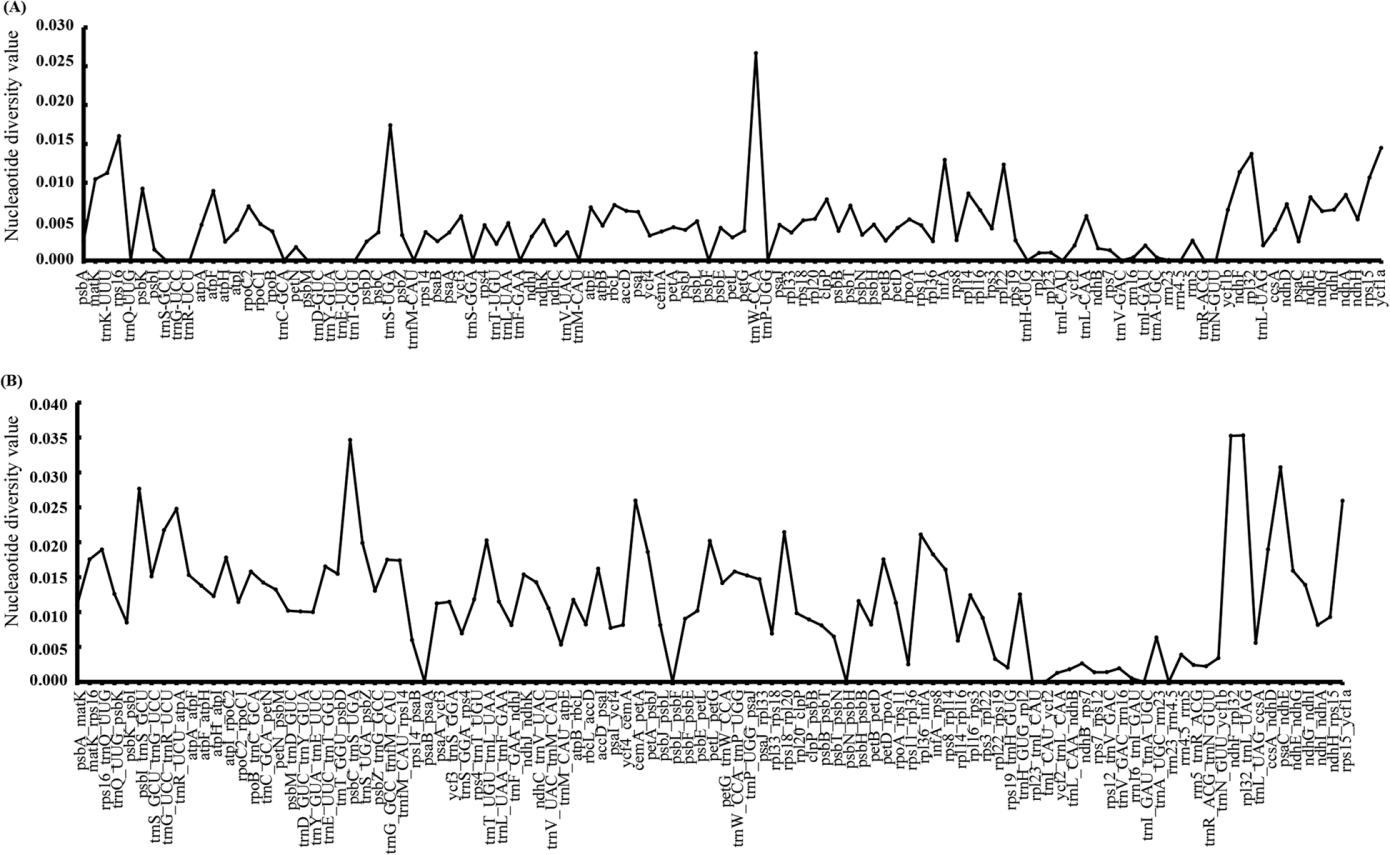
Nucleotide diversity (Pi) values of 111 genes and 103 intergenic regions. (**A**) Nucleotide diversity values of 111 genes. (**B**) Nucleotide diversity values of 103 intergenic regions

We selected 111 genes (Fig. 4 A) and 103 intergenetic regions (Fig. 4 B) to compute their nucleotide diversity (Pi) values by using DnaSP software. As the results indicated, the average Pi value of the genes (0.0043) was smaller than that of the intergenetic regions (0.0118). In terms of Pi values, the top three genes were *trnW-CCA* (0.0266), *trnS-UGA* (0.0174) and *rps16* (0.0160), while the top three intergenetic regions were *rpl32-trnL-UAG* (0.0353), *ndhF-rpl32* (0.0352), and *psbC-trnS-UGA* (0.0346).

### IR/SC borders

**Fig. 5:**
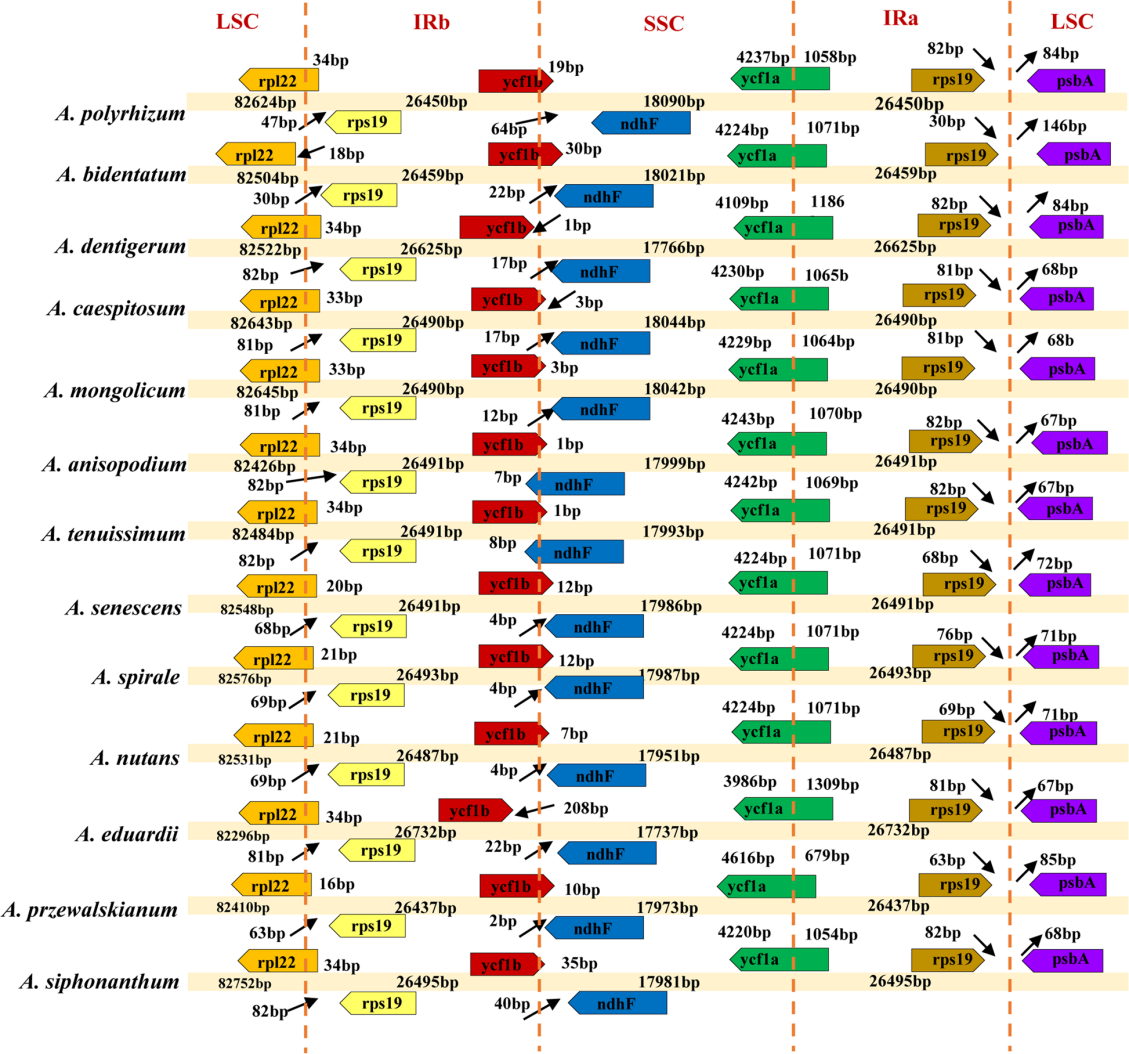
IR/SC boundaries of thirteen *A*. subg. *Rhizirideum* plastomes

Lengths of the IR and SC regions of thirteen *A*. subg. *Rhizirideum* plastomes were compared (Fig. 5). In the results, the longest three IRs belonged to *A.eduardii* (26,732 bp), *A.dentigerum* (26,625 bp) and *A. siphonanthum* (26,495 bp), while the shortest three IRs belonged to *A. przewalskianum* (26,437 bp), *A. polyrhizum* (26,450 bp), and *A. bidentatum* (26,459 bp). For SSC regions, *A. polyrhizum* (18,090 bp), *Allium caespitosum* Siev. ex Bong. et Mey. (18,044 bp) and *A.mongolicum* (18,042 bp) had the top three SSCs, while *A. nutans* (17,951 bp), *A. dentigerum* (17,766 bp) and *Allium eduardii* Stearn (17,737 bp) got the last three ones. The longest three LSCs belonged to *A.siphonanthum* (82,752 bp), *A.mongolicum* (82,645 bp) and *A. caespitosum* (82,643 bp), and the shortest three belonged to *A. eduardii* (82,296 bp), *A. anisopodium* (82,426 bp), and *A. przewalskianum* (82,410 bp).

The positions of IR/SC borders were examined in thirteen *Rhizirideum* plastomes, too (Fig. 5). Gene contents on both sides of the IR/SC borders of thirteen *Rhizirideum* plastomes were conserved. The LSC/IRb border was *rps19*/*rpl22,* and the IRa/LSC border was rps19/psbA. Mostly, *rpl22* was interrupted by LSC/IRb border, and *rps19* was no less than 63 bp away from LSC/IRb border. There were exceptions anyway that the *rpl22* gene of *A. bidentatum* plastome was located entirely in its LSC region (18 bp away from its LSC/IRb border), and the *rps19* gene of *A. polyrhizum* was just 47 bp away from its IRb/LSC border. In *A. bidentatum* plastome, the position of *rps19*/*psbA*, 30 bp/146 bp away from its IRa/LSC border, was also distinguished from others, which were 63 ~ 82 bp and 67 ~ 85 bp, respectively.

For SSC boundaries, two SSC/IR borders crossed two *ycf1* genes in most of the *Rhizirideum* plastomes. Regarding the IRb/SSC border, a large part of the *ycf1b* sequence mainly was located in the IRb region, while gene *ndhF* was completely located in the SSC region. Nevertheless, there were still several exceptions that the whole *ycf1b* gene of the *A. eduardii* plastome was in its IRb region (away from the IRb/SSC border by 208 bp). In plastomes of *A. anisopodium* and *A. tenuissimum*, IRb/SSC borders overlapped *ndhF* genes by 7 bp and 8 bp, respectively. Gene *ycf1*a of most *Rhizirideum* plastomes was 5295 bp in length, except for *A. siphonanthum* (5274 bp), *A. anisopodium* (5313 bp) and *A. tenuissimum* (5313 bp). Gene *ycf1*a was divided into two fragments by border SSC/IRa, and its IRa side ranged from 679 bp (*A. przewalskianum*) to 1309 bp (*A. eduardii*).

### Codon usage bias analysis

**Fig. 6:**
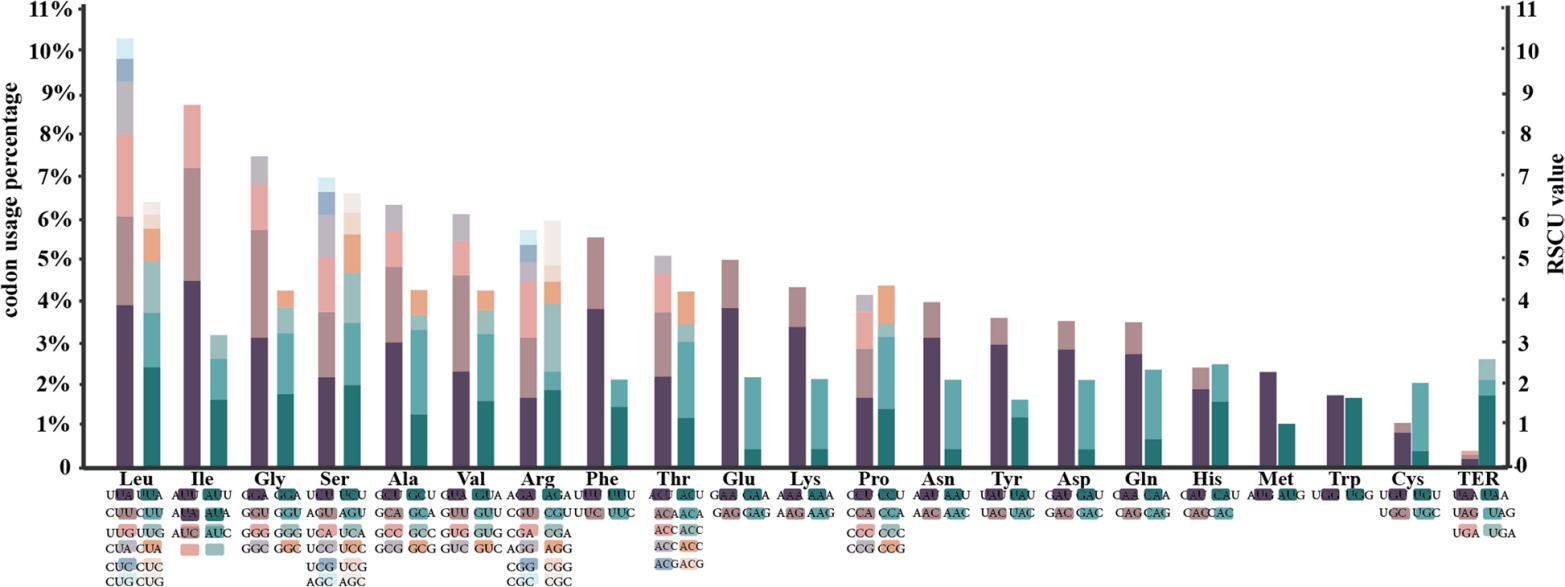
Codon usage in percentages (the left column) and RSCU values (the right column) of twenty-one amino acids. Each codon for an amino acid is shown with different colours

**Fig. 7:**
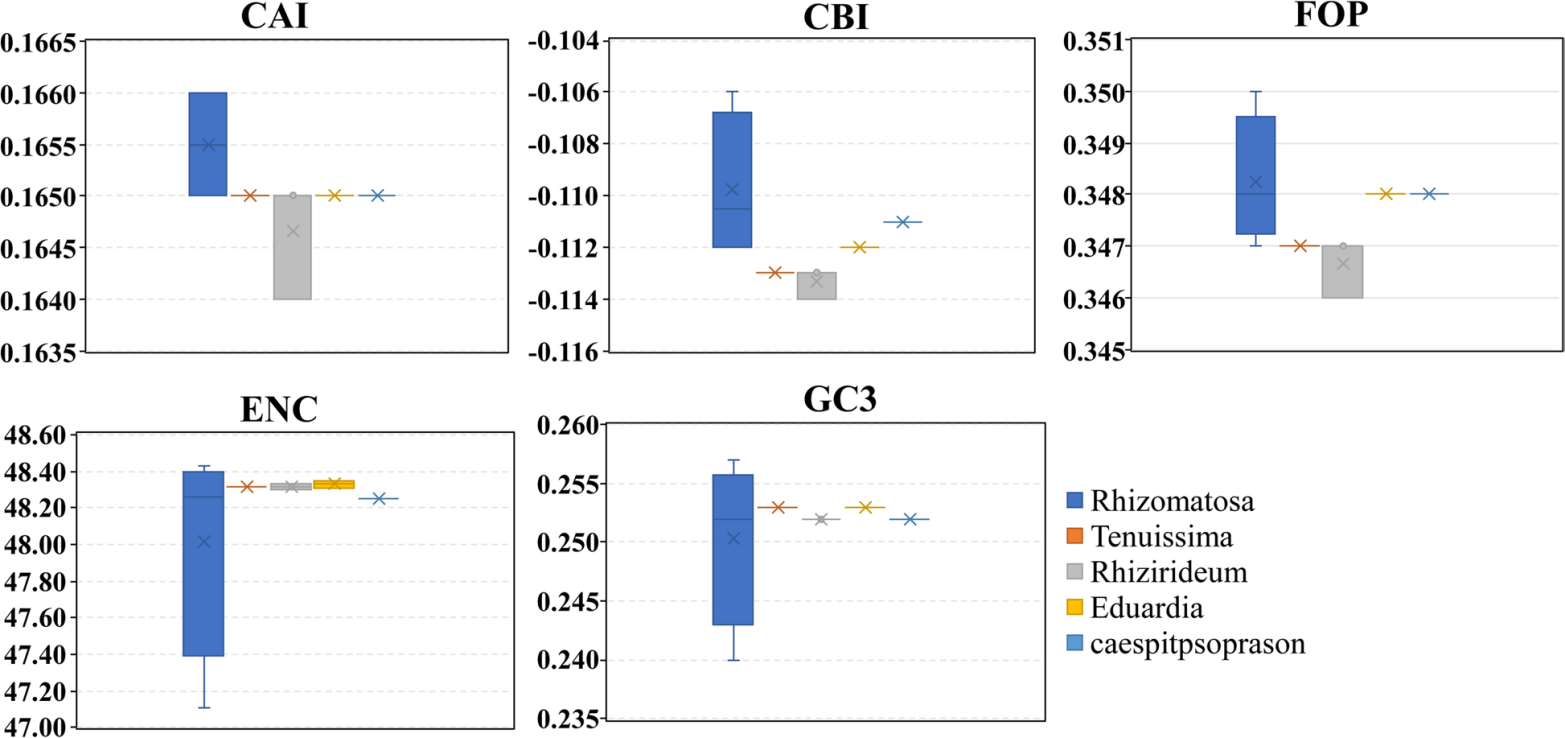
Comparative analysis of codon usage bias in species of five sections in *A*. subg. *Rhizirideum.* CAI, codon adaptation index; CBI, codon bias index; FOP, frequency of optimal codons index; ENC, effective number of codons; GC3: GC percentage of 3rd position in synonymous codons

Seventy-seven protein-coding sequences (CDS) were extracted from thirteen *Rhizirideum* plastomes and were concatenated end-to-end to form a tandem CDS dataset. The codon usage bias of the tandem CDS dataset was analyzed by using program codonW (Fig. 6, Fig. 7, Additional file 6: Table S5 & S6). The total numbers of codons in the CDS tandem sequences ranged from 22,838 (*Allium caespitosum*) to 22,986 (*Allium eduardii*). The average values of relative synonymous codon usage (RSCU) of each sort of codon in thirteen tandem CDS sequences ranged from 2.08 (UUA) to 0.31 (CUG, AGC). In terms of codon proportions, six synonymous codons coding leucine (Leu) accumulated to a largest proportion of 10.35%, and two codons coding cysteine (Cys) accumulated to a smallest proportion of 1.13% except for three stop codons (0.40%). Methionine (Met, AUG) and tryptophan (Trp, UGG) showed no codon bias and were encoded by only one codon. Thirty codons with RSCU > 1 encoded nearly all kinds of amino acids except for Trp and Met. Among the thirty codons, only UUG for Leu was ended by G/C, and the last twenty-nine were ended by A/U.

### Repeat sequence analysis

**Fig. 8:**
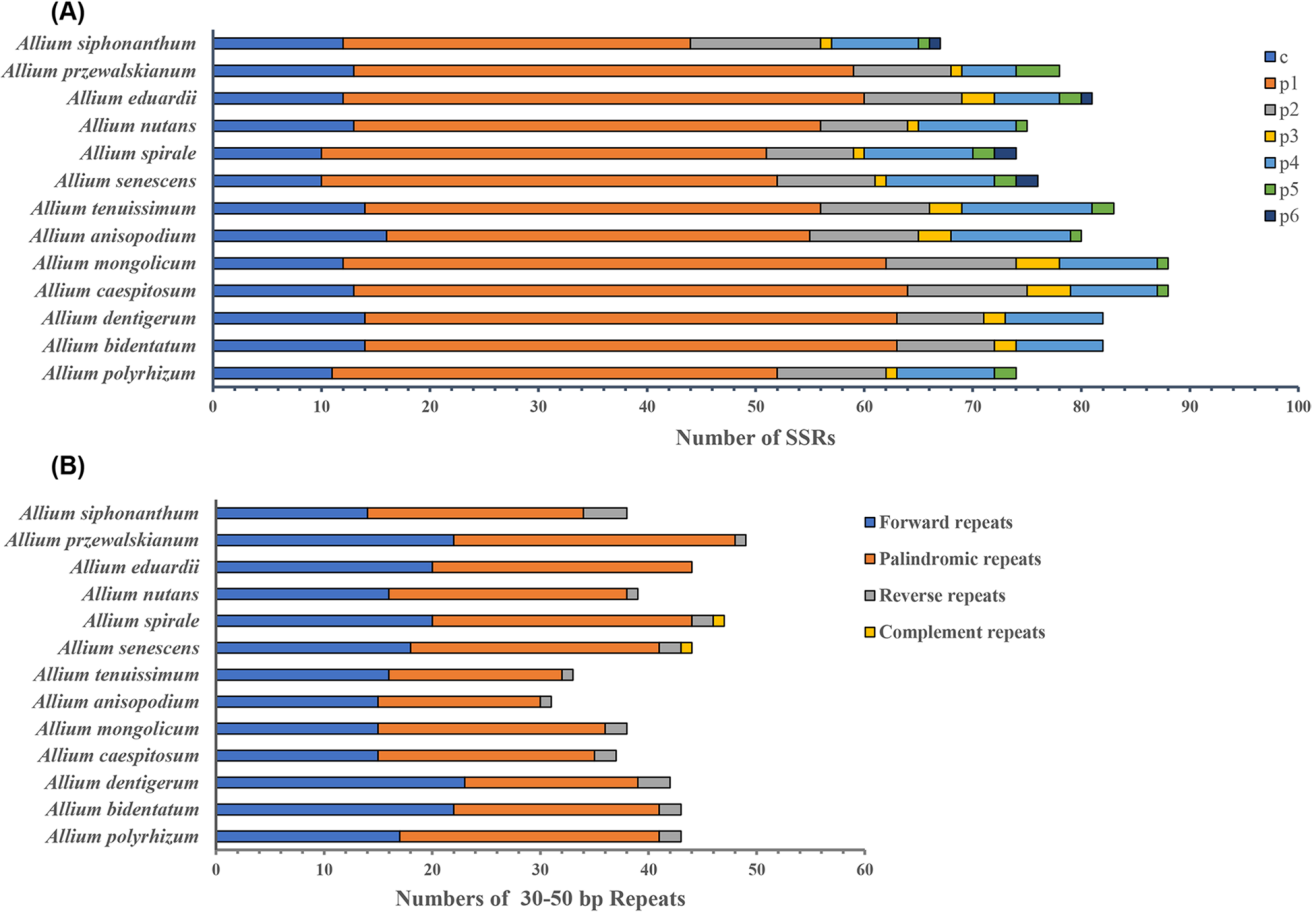
Numbers of SSRs and repeats of thirteen *A.* subg*. Rhizirideum* species. (**A**), stacking histogram of SSR numbers. Seven sorts of SSRs are shown with different colors: c, compound microsatellites; p1-p6, microsatellites with one to six bases as a repeat unit. (**B**), stacking histogram of repeats (30–50 bp) numbers

We detected 879 simple sequence repeats (SSRs) in thirteen *Rhizirideum* plastomes (Fig. 8 A). *A. mongolicum* and *A. caespitosum* contained the most SSRs (88) whereas *A.siphonanthum* contained the least (67). SSRs with 1 bp ~ 5 bp could mostly be witnessed across thirteen plastomes, but those with 6 bp (i.e., the hexanucleotides) were rare, only existing in four of the plastomes (*A.senescens* 2, *A.spirale* 2, *A.eduardii* 1, *A.siphonanthum* 1). Among all kinds of SSRs of all thirteen plastomes, mononucleotides (55.74%) were the most abundant, followed by compound microsatellites (15.95%), dinucleotides (12.16%), tetranucleotides (11.09%), trinucleotides (2.63%), pentanucleotides (1.99%) and hexanucleotides (0.58%). Most of the SSRs were composed of A/T while G/C rarely occurred. Furthermore, SSRs were distributed more in LSC regions than in IR or SSC regions.

In addition to SSRs, repeats of 30 bp ~ 60 bp were also detected (Fig. 8 B). Four types of repeats were summed up to 528, including forward, reverse, palindromic and complementary. The proportion of palindromic repeats (51.14%) was the highest, while that of the complementary repeats (0.38) was the lowest. *A. przewalskianum* contained the most repeats (49), and *A. anisopodium* contained the least (31).

### Phylogenetic analysis in subgenus *Rhizirideum*

**Fig. 9:**
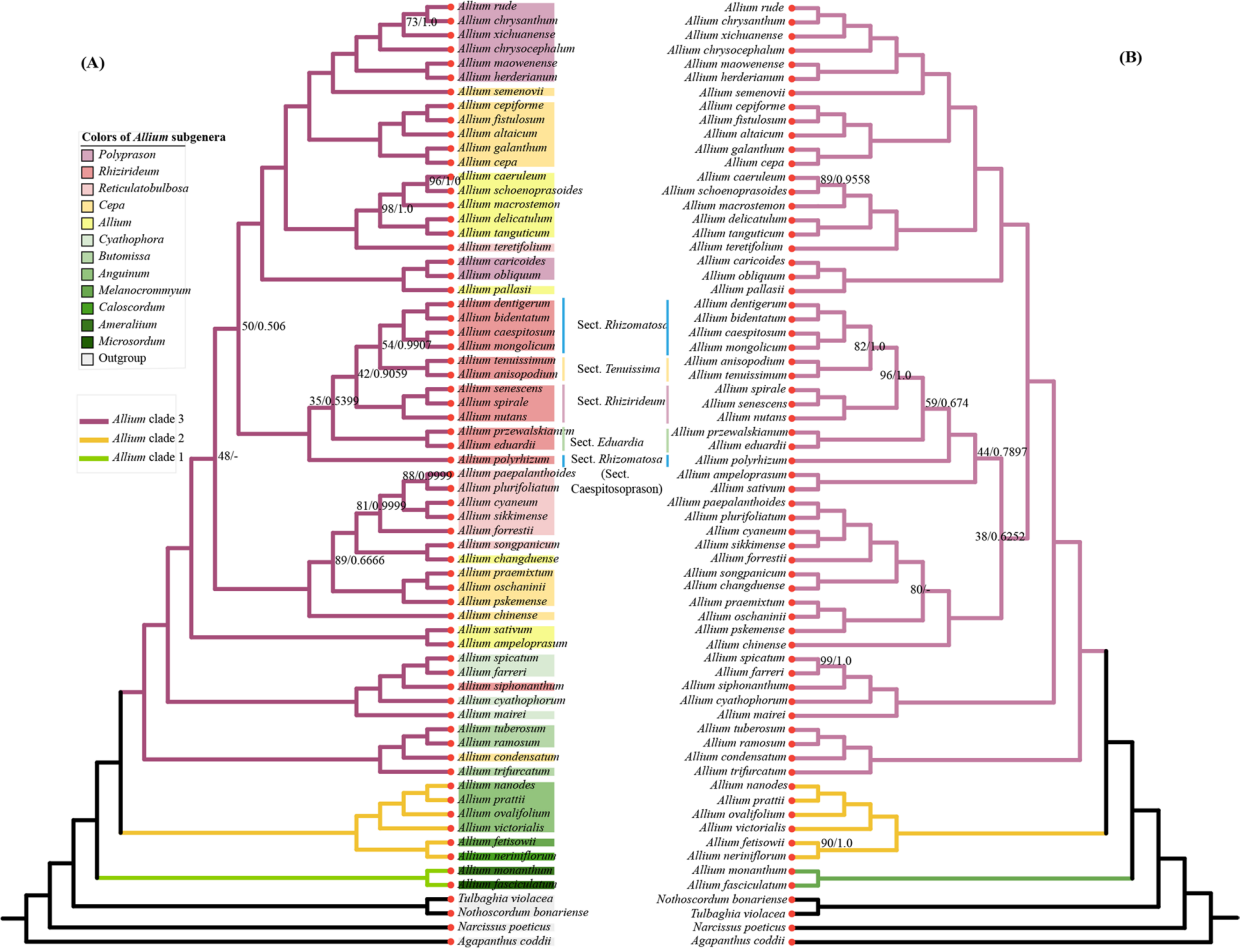
Phylogenetic tree reconstruction of 67 species inferred from Maximum likelihood (ML) and Bayesian inference (BI) analyses based on CDS sequences and complete plastomes. (**A**), CDS tree. (**B**), complete plastome tree. Tip colors, colors of subgenus names of genus *Allium* on the clade. Branch colors, colors of three evolutionary clades of genus *Allium*. The bootstrap support values are listed on the left side of slash (/) and posterior probability values are listed on the right side of slash (/). Null means 100% or 1. The minus sign (−) means parallel branch in BI tree

Seventy-seven protein-coding sequences of sixty-seven plastomes were extracted and concatenated to establish a tandem CDS dataset. The complete chloroplast genomes (cp) of sixty-seven species were multiple-aligned and trimmed to establish a cp dataset. The CDS dataset and the complete chloroplast genome (cp) were used to reconstruct phylogenetic trees. The CDS tree (Fig. 9 A) and the cp tree (Fig. 9 B) showed a similar topology. Thus, we will take the CDS tree as an example to explain the intra-subgenus (*A*. subg. *Rhizirideum*) and inter-subgenera relationships, and the differences between the CDS tree and the cp tree will be explained at the end of this section.

Within *A*. subg. *Rhizirideum* clade, there are twelve species clustered into five small clades. *A. anisopodium* and *A. tenuissimum* formed a clade and belonged to *A*. sect. *Tenuissima*. Species *A. caespitosum*, *A. mongolicum*, *A. bidentatum* and *Allium dentigerum* Prokh. formed a clade, and belonged to *A*. sect. *Rhizomatosa*. Species *A. senescens*, *A. spirale* and *A. nutans* clustered to form *A*. sect. *Rhizirideum*, and *A. eduardii* and *A. przewalskianum* formed *A*. sect. *Eduardia*. Interestingly, *A. polyrhizum*, previously belonging to *A*. sect. *Caespitosoprason*, was resolved as a sister to clade *A*. sect. *Tenuissima* + *A*. sect. *Rhizomatosa* + *A*. sect. *Rhizirideum* + *A*. sect. *Eduardia.* Besides, *A. siphonanthum*, previously belonging to *A*. sect. *Eduardii*, was now resolved as a sister to *A. spicatum* + *A. farreri* in clade *A*. subg. *Cyathophora*.

Within genus *Allium* (Fig. 9 A), there was a distinct division of three evolutional clades. For the eleven subgenera involved in this study, *A*. subg. *Microsordum* and *A*. subg. *Amerallium* formed clade 1, *A*. subg. *Caloscordum, A.* subg. *Melanocrommyum* and *A.* subg. *Anguinum* formed clade 2, and the left seven subgenera formed clade 3. In clade 3, only *A.* subg. *Rhizirideum* is monophyletic while the other six subgenera are polyphyletic (*A*. subg. *Polyprason, A*. subg. *Cepa*, *A*. subg. *Allium, A*. subg. *Reticulatobulbosa, A*. subg. *Cyathophora,* and *A*. subg. *Butomissa*). Six species in *A*. subg. *Polyprason* (*A.rude, A.chrysanthum, A.xichuanense, A.chrysocephalum, A.maowenense, A.herderianum*) were clustered with six *A*. subg. *Cepa* species (*A. cepa, A. galanthum, A. altaicum, A. fistulosum, A. cepiforme, A. semenovii*) by a support ratio of 100/1.0, then clustered with a branch of five *A*. subg. *Allium* species (*A.caeruleum, A.schoenoprasoides, A.macrostemon, A.delicatulum, A. tanguticum*) + one *Reticulatobulbosa* species (*A. teretifolium*) by 100/1.0. Two species from *A*. subg. *Polyprason* (*A.caricoides, A.obliquum*) and one *A*. subg. *Allium* species (*A. pallasii*) formed a small branch (100/1.0) and then became a sister to Clade *A*. subg. *Polyprason + A*. subg. *Cepa + A*. subg. *Allium* (100/1.0). And *A*. subg. *Rhizirideum* was resolved as a sister to the branch just mentioned (50/0.506). Five *A*.subg. *Reticulatobulbosa* species (*A. paepalanthoides, A. plurifoliatum, A.cyaneum, A.sikkimense, A.forrestii*) formed a branch (81/0.9999) and then clustered with a small clade of one *A*. subg. *Reticulatobulbosa* species (*A. songpanicum*) *+ A. changduense* from *A*. subg. *Allium* (100/1.0). This branch was clustered with a clade of three *A*. subg. *Cepa* species (*A. praemixtum, A. oschaninii, A. pskemense*) (89/0.6666), then clustered with another *A*. subg. *Cepa* species, *A. chinense* (100/1.0). Clade *A*. subg. *Reticulatobulbosa + A*. subg. *Cepa* was resolved as a sister to the Clade *A*. subg. *Polyprason + A*. subg. *Cepa + A*. subg. *Allium + A*. subg. *Rhizirideum* (48/−). Clade *A*. subg. *Reticulatobulbosa + A*. subg. *Cepa* was a parallel clade to the Clade *A*. subg. *Polyprason + A*. subg. *Cepa + A*. subg. *Allium + A*. subg. *Rhizirideum* in the BI CDS tree. Then two another *A*. subg. *Allium* species (*A. sativum, A. ampeloprason*) formed a sister to the big clade just mentioned. And one another *A*. subg. *Cepa* species (*A. condensatum*) was resolved in the *A*. subg. *Butomissa* clade.

In cp tree, there is several differences in topology. The Clade *A*. subg. *Reticulatobulbosa +* three species from *A*. subg. *Cepa* (*A. praemixtum, A. oschaninii, A. pskemense*) possessed a support ratio of 80/−, which means a parallel clade in BI tree, while the ratio of CDS tree came to 89/0.6666. The clade of two *A*. subg. *Allium* species (*A. sativum, A. ampeloprason*) was resolved as a sister to clade *A*. subg. *Rhizirideum* by 44/0.7897, and this big clade was then clustered to the mentioned clade *A*. subg. *Reticulatobulbosa + A*. subg. *Cepa* by 38/0.6252. And the Clade *A*. subg. *Rhizirideum + A*. subg. *Allium* + *A*. subg. *Reticulatobulbosa + A*. subg. *Cepa* was resolved as a sister to clade *A*. subg. *Polyprason + A*. subg. *Cepa + A*. subg. *Allium* by 100/1.0.

### Gene selective pressure

We calculated the Ka/Ks ratio (ω) of seventy-seven common protein-coding sequences (CDSs) in thirteen *Rhizirideum* plastomes (Fig. 9) and then estimated the selective pressure (Additional file 9: Table S9). Most ω values were less than 1, while three CDSs were found 0.5 < ω < 1.0 (*rbcL, ycf1a, ycf1b*) and one ω > 1 (*ycf2*) (Fig. 9). Unexpectedly, after selective pressure analysis in EasyCodeMl, none of the seventy-seven CDSs was found significant (*P* < 0.05) after the likelihood ratio test (LRT). We examined the functions and relative biochemical pathways of the four protein-coding genes mentioned above (Table 3).

**Table 3:** Genes under positive and relaxed selection in *A*. subg. *Rhizirideum* plastomes

Gene	Ka/Ks ratio	Selection pressure	Gene description	Pathway
*ycf2*	1.107	Positive selection	Ycf2, part of a 2-MD heteromeric AAA-ATPase complex	Photosynthesis
*rbcL*	0.563	Relaxed selection	ribulose-1,5-bisphosphate carboxylase/oxygenase large subunit	Calvin-Benson Cycle
*ycf1a*	0.787	Relaxed selection	Tic214, part of a translocon at the inner envelope membranes of chloroplast called TIC	Photosynthesis
*ycf1b*	0.544	Relaxed selection	Tic214, part of a translocon at the inner envelope membranes of chloroplast called TIC	Photosynthesis

## Discussion

### Comparative plastome structure analysis of *A*. subg. *Rhizirideum*

Although events of evolution such as genome rearrangement, gene loss, IR expansion, and contraction, have been detected for many times, plastomes are generally highly conserved in genome size, structure, and gene content [32–40]. In this study, the *A*. subg. *Rhizirideum* plastomes are of high conservation by large. The quantity of genes, CDSs, rRNA-coding genes, and tRNA-coding genes is 141, 87 (or 89), 8, and 38, respectively, which follows most angiosperms [28, 36, 38, 40–42].

There were 5 of 141 genes pseudogenized (*orf*56, *ycf*15, *rps*2, *inf*A, *ycf68*) (Table 4). Plastome genes *ycf15*, *ycf68*, and *infA* are also pseudogenized in many other species such as *Malus pumila*, *Morus alba*, *Cynodon dactylon* [38, 41, 43, 44]. The *rps2* gene, encoding ribosomal protein S2, is lost in *A*. sect. *Daghestanica* plastomes but is pseudogenized in *Chlorophytum rhizopendulum* [30, 44]. In addition, *rps2* production is of great significance to the defense signal transduction process [45]. Thus, in terms of genes coding confirmed products (*infA* & *rps*2), their pseudogenization might be used to adjust the transcription and signal transduction of *Rhizirideum* plants in response to the changing environment.

**Table 4:** Summary of pseudogenes and their productions in* A.* subg. *Rhizirideum* plastomes

Pseudogene	Position	Production
*ycf68*	IR	Putative protein RF68
*orf56*	IR	Putative protein RF56
*ycf15*	IR	Putative protein RF15
*rps2*	LSC	Ribosomal protein S2
*infA*	LSC	Translation initiation factor 1

The SC/IR borders of angiosperm plastomes are generally conservative, lying mostly beside *rps19* and *ycf1* [46]. Genes *trnH-GUG* and *trnN-ACG* are believed to be located at the IR/LSC and IR/SSC borders of the ancestor of monocots, respectively [37]. According to the relative positions of *rps19*/*trnH-GUG* and *ycf1*/*trnN-ACG* in* A. *subg. *Rhizirideum* plastomes (Fig. 2), an expansion of IR regions might occurred. Generally speaking, expansion of IRs can lead to the movement of SC/IR borders. Most terrestrial plants, as *A*. subg. *Rhizirideum* species, present movements to a tiny extent, which can make a few genes into or out of IRs [47–49]. Nonetheless, some plants do have their IRs expanding in a large scale. The large expansion can contribute to a large increase or loss of IR genes, such as species in *Pelargonium*, *Psilotum*, *Leguminosae,* and *Erodium* [32, 33, 50–55]. In *A*. subg. *Rhizirideum* plastomes, the duplicated *rps19* moved into the IRs from the LSC, while the incompletely duplicated *ycf1* moved to cover the IR/SSC borders from the SSC (Fig. 5). In addition, the LSC/IRb boundaries also present a slight shift to the *rpl22* gene. The movements of IR/SC borders of *A.* subg. *Rhizirideum* plastomes are tiny compared to the species mentioned above. Despite this, the IR expansion of our taxa is somewhat significant for the evolution. It is known that IR regions possess the nature of self-duplication, which has been proven to reduce the synonymous mutation rate (Ks) of genes, resulting in the Ks of IR genes being generally lower than that of SC genes [48]. It can be inferred that in the* A*. subg. *Rhizirideum* plastomes, the Ks value of the *rps19* gene decreased after moving from the LSC to the IRs. That is, the *rps19* gene has been more conserved, as well as its product, ribosomal protein S19, which is a component of the 40S ribosomal subunit. Therefore, it may contribute to the increase of stability of the ribosomal structure when *rps19* moved to IRs. This is also true of the gene *rpl22* coding ribosomal protein L22, a component of the 60S ribosomal subunit. The moving trend of *rpl22* may also influence the ribosomal structure. As is known, the structure of ribosomes can influence the expression pattern of genes, which are often relative with the environment [56]. Consequently, the shift of IR/SC boundaries may be regarded as the adaptive evolution of plastomes. There are twenty-six genes with introns in the plastome of *A. senescens*, three more than *Anena sativa* in the family *Gramineae* (single-copy gene *clp*, *rpoC*1 and double-copy gene *ycf*68). The transpliced gene *rps12* has three exons, one in the LSC and two in the IRs. In eukaryotes, intron-splicing enhances gene expression by reducing transcription-associated mutagenesis [57]. Meanwhile, this process imposes selection pressure on genes [58]. Therefore, the intron-existing genes in *A*. subg. *Rhizirideum* plastomes indicate that they are also under this kind of pressure.

The GC contents of *A*. subg. *Rhizirideum* plastomes range from 36.8 to 36.9%, which is in accordance with those of many other monocots, approximately 37% [59–61]. Additionally, the GC content of Amarillydaceae subfamily *Allioideae* plastomes is below that of other families, such as Asparagaceae, Iridaceae, Agapanthaceae, Etc. [62]. This decrease can be attributed to the selective pressure caused by either neutral mutation [63–65] or high transcription efficiency [66, 67]. This is the same as the low GC content of the* A.* subg. *Rhizirideum* plastomes.

Codon usage bias is a significant feature of plastomes, which influences gene expression and demonstrates natural selection pressure [68, 69]. According to the results, we found that subgenus *Rhizirideum* have thirty frequently used codons (RSCU > 1), 29 of which ended by A or U. In plastomes, codons often appeared with a higher AU content. The third position of codons have a higher trend of using A/U than G/C [70–72]. Codons encoding leucine were the most of all, and the codon bias showed as UUA > CUU > UUG > CUA > CUC > CUG, consistent with other plants like *Ligusticum* and *Geraniaceae* [40, 73]. From Fig. 7, we found that differences in CAI, ENC and GC3 of five *A.* subg. *Rhizirideum* sections were small, while differences in CBI and FOP were relatively more significant. CBI and FOP of section *Rhizomatosa* had the highest values and those of section *Rhizirideum* had the lowest. The results revealed that the diversity of codon usage patterns of different taxa might also be helpful for the identification and classification of species [74].

SSRs are regarded as potential resources in evolutionary research and are effective in species classification and population genetic analyses that study the biogeography of allied taxa [75–79]. According to the SSR counting results (Fig. 8 A, Additional file 7: Table S7), we found some repeats only in some species, such as hexanucleotides TTTCCC in *A. siphonanthum*, pentanucleodide TTTAG in *A. przewalskianum,* and trinucleotides CTT in *A. mongolicum* and *A. caespitosum*. These unique SSRs can be used for species identification and classification in subgenus *Rhizirideum*. There have been SSRs detected for this purpose, like *Lycoris*, *Psidium*, and *Asparagus* [80–82]. Thus, we hope SSRs detected in our study will provide some helpful information for research of *Allium* in the future. Besides, large repeat sequences can promote plastome rearrangement and play an important part in sequence divergence [83–85]. In our study, 528 repeats of 30–50 bp were detected (Fig. 8 B). Among all kinds of large repeats, forward repeats and palindromic repeats were in the majority, similar to many other species [40, 86, 87]. Moreover, we found that complement repeats were specially owned by *A. spirale* and *A. senescens*.

### Phylogenetic analysis

Appropriate gene combinations are significant for accurate phylogenetic inference. Nuclear DNA genes (e.g., ETS and ITS), cpDNA fragments (e.g.,*matK, trnL-trnF,* and *psbJ-petA*) and plastomes have been used for the phylogenetic estimation of plants. Here, we used two datasets (complete chloroplast genomes and chloroplast CDSs) to conduct ML analysis and BI analysis for the reconstruction of *A*. subg. *Rhizirideum* phylogeny. According to previous studies based on ITS sequences, *Allium* species were divided into three lineages called clade 1, clade 2 and clade 3 [8]. In a study based on ITS [8], the subgenera *Cepa, Reticulatobulbosa, Polyprason*, and *Allium* formed parallel branches, which clustered with *Rhizirideum*. The results of a recent study based on plastomes [31] showed that *A*. subg. *Cepa* first clustered with *A*. subg. *Polyprason*, then successively clustered with *A*. subg. *Allium*, small branch of *A*. subg. *Polyprason* species, small branch of *A*. subg. *Allium* species, another *A*. subg. *Cepa* branch, and *A*. subg. *Rhizirideum*. These previous studies indicated that the subgenera *A*. subg. *Cepa*, *A*. subg. *Reticulatobulbosa*, *A*. subg. *Polyprason*, and *A*. subg. *Allium* were polyphyletic groups. Concerning the inter-subgenus relationships within the genus *Allium*, the topology of our phylogenetic trees (Fig. 9) are generally consistent with previous studies based on ITS and plastomes [9, 23, 88]. Phylogenetic analysis results (Fig. 9) demonstrate that *A*. subg. *Rhizirideum* is a strongly supported monophyletic group, which corresponds with previous reports [8]. However, other subgenera near *A*. subg. *Rhizirideum*, like *A*. subg. *Allium* and *A*. subg. *Cepa*, are polyphyletic groups. For instance, several species from *A*. subg. *Cepa* are clustered with *A*. subg. *Reticulatobulbosa* clade (*A. praemixtum, A. oschaninii, A. pskemense and A. chinense*) and *A*. subg. *Butomissa* clade (*A. condensatum*), and species from *A*. subg. *Allium* are clustered with *A*. subg. *Reticulatobulbosa* clade (*A. changduense*). More genomic samples and geographic information are required for further investigation in the future.

In a phylogenetic tree based on ITS-rps16 datasets [9], *A*. sect. *Rhizomatosa* clustered with *A*. sect. *Caespitosoprason*, and *A*. sect. *Tenuissima* clustered with *A*. sect. *Rhizirideum*. The mentioned two clades were resolved as sister branches and clustered with *A*. sect. *Eduardia*. In our results (Fig. 9), clade *A*. subg. *Rhizirideum* has five branches containing 12 of our 13 species (except *A. siphonanthum*) and each of them represents a section of this subgenus (*A*. sect. *Rhizomatosa, A*. sect. *Tenuissima, A*. sect. *Rhizirideum, A*. sect. *Eduardia,* and *A*. sect. *Caespitosoprason*). Species in *A*. sect. *Eduardii* (*A. przwalskianum* & *A. eduardii*) and *A*. sect. *Tenuissima* (*A. anisopodium* & *A. tenuissimum*) cluster into two individual branches, which is the same as the phylogenetic analysis of Li et al. [9]. Nevertheless, the interspecific relationships of the other two sections, *A*. sect. *Rhizirideum* and *A*. sect. *Rhizomatosa*, are somewhat different. The section *A*. sect. *Rhizirideum* was divided into Asiatic and European geographical groups by Sinitsyna et al. [24] and the species *A. senescens*, *A. spirale* and *A. nutans* were in the former group, but the relationships among *A. senescens*, *A. spirale* and *A. nutans* were not so clearly shown. Our results show that *A. senescens* and *A. spirale* form a sister branch and then cluster with *A. nutans* with a 100/1.0 support rate. Friesen et al. [10] conducted a phylogenetic analysis for species in two *A*. subg. *Rhizirideum* sections (*A*. sect. *Rhizomatosa*, and *A*. sect. *Caespitosoprason*) based on chloroplast DNA fragments, where *A*. sect. *Caespitosoprason*, including *A. polyrhizum,* was merged into *A*. sect. *Rhizomatosa*. In contrast, our plastome tree indicates that *A. polyrhizum* is not clustered with *A*. sect. *Rhizomatosa* species but with clade *A*. sect. *Rhizomatosa + A*. sect. *Tenuissima + A*. sect. *Rhizirideum + A*. sect. *Eduardia* in *A*. subg. *Rhizirideum*. In other words, *A. polyrhizum* may be separated from *A*. sect. *Rhizimatosa* and placed back into *A*. sect. *Caespitosoprason,* which is believed to be a basal taxon of this subgenus. Additionally, *A. siphonanthum*, a member of *A*. sect. *Eduardia,* is now clustered with *A*. subg. *Cyathophora*. *A. siphonanthum* characters as umbel densely many flowered, pedicels shorter than perianth, and bulb tunic subreticulate, while *A. cyathophorum* in *A*. subg. *Cyathophora* is charactered as umbel laxly flowered, Pedicels 1–3 times as long as perianth, and bulb fibrous sometimes subreticulate. So there is few similarity between *A.siphonanthum* and *Cyathophora* species. This may be an example of disagreements between molecular and morphological analyses, but identifying the phylogenetic position of *A. siphonanthum* still needs more specimens and molecular evidence.

Molecular relationships are often consistent with morphology characteristics. Except for *A*. sect. *Caespitosoprason*, the rest four clades of *A*. subg. *Rhizirideum* can represent the four typical phenotypes of this subgenus (Fig. 9, Fig. 1, Additional file 1: Fig. S1). Species in *A*. sect. *Rhizomatosa* character as leaf semiterete to terete, bulbs densely clustered, and the outer skin fibrous. *A*. sect. *Tenuissima* species character as leaf semiterete, bulb clustered, and outer skin not broken. *A*. sect. *Eduardia* species character as leaf semiterete, and bulbs covered with a common reticulate tunic. Species in *A*. sect. *Rhizirideum* have a very different morphology from other sections, leaf broadly linear and bulb ovate-cylindric and thicker. Despite this, one species in *A*. sect. *Caespitosoprason* (*A. polyrhizum*) and four species in *A*. sect. *Rhizomatosa* (*A. caespitosum*, *A. bidentatum*, *A. mongolicum*, and *A. dentigerum*) are relatively similar in morphology. Disagreements between molecular and morphological analyses have also been frequently reported in other taxa, for instance, section *Daghestanica* and subgenus *Cyathophora* in the genus *Allium* [30, 89].

As is shown in Fig. 9, intra-section relationships in five sections of *A*. subg. *Rhizirideum* are supported by high support (100/1.0). However, the inter-section relationships among some sections show a lower support ratio, such as the node between *A*. sect. *Rhizomatosa* and *A*. sect. *Tenuissima* (54/0.9907 in CDS tree, 82/1.0 in cp tree). This phenomenon probably occurs because of lacking samples. Both bootstrap support values and posterior probabilities in cp tree (Fig. 9 B) are relatively more prominent than those in CDS tree, especially the node between *A*. sect. *Eduardia* and clade *A*. sect. *Rhizomatosa + A*. sect. *Tenuissima + A*. sect. *Rhizirideum* (35/0.5399 in CDS tree, 59/0.674 in cp tree). This may be due to the fewer genetic sites in CDSs than those in cpDNA sequences. Also, the numbers of support ratio in ML tree are smaller than in BI tree, possibly because of the difference in inference methods.

### Adaptive evolution

**Fig. 10:**
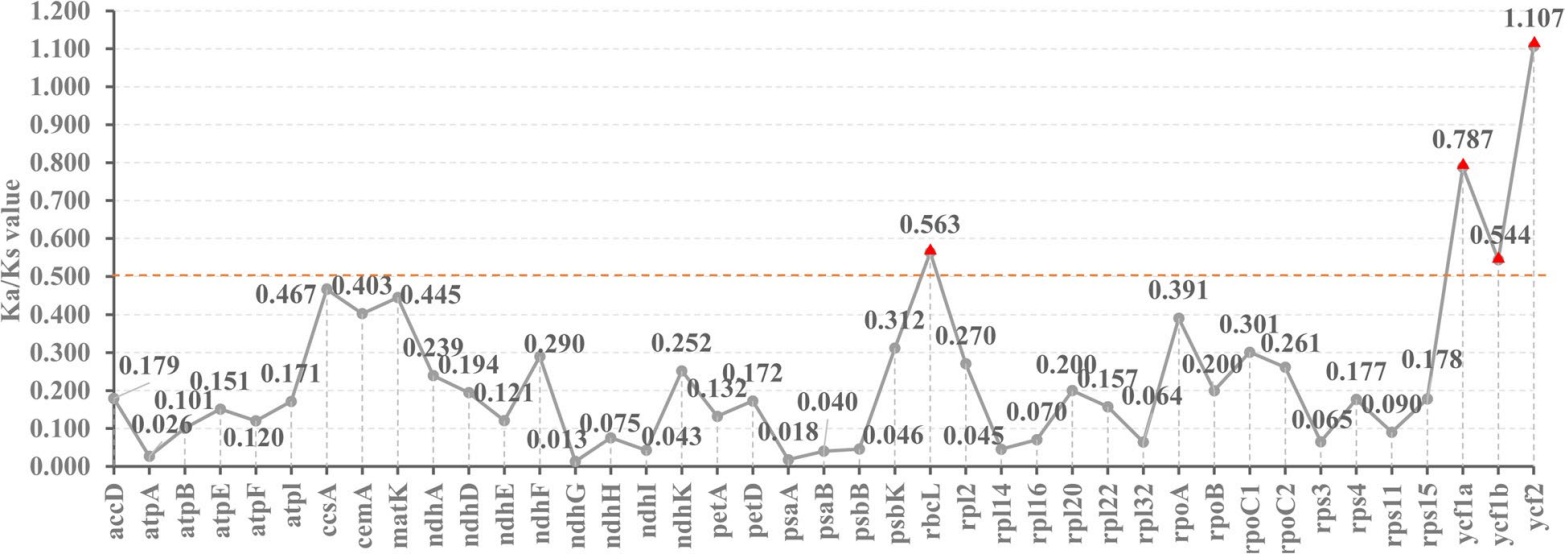
Ka/Ks ratios of 41 single-copy genes. Three genes > 0.5 and one gene > 1 are noted with red triangles

The Ka/Ks ratio (ω) is used to assess the selective pressure on protein-coding genes. The ω values > 1, = 1, and < 1 indicate that this gene has undergone positive, neutral, and purifying selection, respectively. In addition, there is also a sort of relaxed selection with 0.5 < ω < 1, according to other research [30, 40, 62]. The Ka/Ks calculating results (Fig. 10) showed that most of the genes had a ω < 0.5, while one of the genes had a ω > 1 (*ycf2*) and three of them had a ω > 0.5 (*rbcL, ycf1a, ycf1b*). So we consider that *ycf2* has been under positive selection and *rbcL* and *ycf1* have been under relaxed selection. In previous studies, those four genes mentioned above have been reported under positive selection [90–94]. Gene *rbcL* encodes ribulose-1,5-bisphosphate carboxylase/oxygenase (RuBisCO) large subunit that is vital to CO_2_ fixation by plants. RuBisCO catalyzes the production of 3-phosphoglycerate by adding CO_2_ onto RuBP, which provides the resource for sugar synthesis [95]. Genes *ycf1* and *ycf2* have been enigmatic and their functions had not been found for a long time until knockout studies showed that the gene *ycf1* is essential for the survival of plants [96–98]. The latest study has proved that *ycf1* encodes a protein Tic214 that forms a vital component of a translocon at the inner envelope membranes of chloroplast called TIC, which is indispensable for photosynthetic protein import in green tissues [99]. The gene *ycf2* has also been proven to encode a protein, part of a 2-MD heteromeric AAA-ATPase complex, which is closely associated with the TIC complex and functions as a motor for protein import [100]. Thus, these genes with a ω > 0.5 are necessary for photosynthesis, which is essential for plants. Species of *A*. subg. *Rhizirideum* are distributed in extreme environments, such as areas with very low temperature, arid climates, and high altitudes [10, 24, 101]. The photosynthesis demands for sufficient light might have exerted relatively intensive pressure on these genes. The other way round, the positively or relaxedly selective genes may help those species fit in the various environments.

## Methods

### DNA isolation, sequencing and plastome annotating

The fresh leaves of eleven species were sampled from public areas and dried with silica gel afterwards (locality see Additional file 5: Table S4). Total genomic DNA was isolated from silica-dried leaf tissues with a modified CTAB method. The voucher specimens (Additional file 5: Table S4) were deposited at the herbarium of Sichuan University (Chengdu, China) (voucher specimens: H11072607 (SZ), De-qing Huang; ZCJ20210821 (SZ), Chun-jing Zhou; FX2020081001 (SZ), Xiao Fu; FX2020080902 (SZ), Xiao Fu; H11072807 (SZ), De-qing Huang; H11070501 (SZ), De-qing Huang; FX2020081401 (SZ), Xiao Fu; FX2020081501 (SZ), Xiao Fu; FX2020081901 (SZ), Xiao Fu; ZCJ2012081910 (SZ), Chun-jing Zhou; FX2021072101 (SZ)). And the DNA sample of *A. siphonanthum* was from Germplasm Bank of Wild Species and National Wild Plant Germplasm Resource Center, voucher specimen 13CS6776 (KUN) at Kunming Institute of Botany. DNA libraries were prepared and sequenced with the Illumina HiSeq 2500 platform with PE150 bp reads.

Complete chloroplast genomes were reconstructed by NOVOPlasty v2.6.2 [102] using *A. cepa* (MK335926) and *A. sativum* (MK335928) as references. Then the plastid genomes were annotated with PGA [103] and manually adjusted with Geneious R11 (Biomatters, Ltd., Auckland, New Zealand). Finally, the plastome circus map was drawn with OGDRAW [104] and Gview [105].

### Synonymous codon usage bias

Seventy-seven protein-coding sequences (Additional file 3: table S2) were extracted from thirteen *Rhizirideum* plastomes with Phylosuite v1.2.2 [106], aligned with MAFFT v7.487 [107] and trimmed with trimAl v1.2 [108]. Then again with Phylosuite, they were concatenated respectively and form thirteen CDS tandem sequences. Afterwards, the thirteen sequences were input into codonW v1.3 to calculate codon contents and RSCU values, which were later sorted and analyzed manually in Microsoft Excel 365.

### Sequence divergence

The online program mVISTA [109] was used to generate the whole-genome alignment of the thirteen *Rhizirideum* plastomes with *A. senescens* as a reference. All the plastomes were aligned with MAFFT v7.487. The nucleotide diversity (Pi) of genes and intergenic regions was calculated by DnaSP v6 [110].

### Repeat structure

REPuter [111] was used to examine plastome repeat sequences. Thirteen plastomes of subgenus *Rhizirideum* were input into the REPuter website and the list of repeats were exported. Four sorts of repeats were classified: forward, palindromic, reverse, and complimentary matches. The parameters were as follows: repeat size of (1) > 30 bp; (2) > 90% sequence identity between the two repeats; and (3) Hamming distance = 3. Simple sequence repeats (SSRs) of thirteen A. subg. *Rhizirideum* plastome sequences were mass counted by Perlscript MicroSAtellite (MISA). The setting motif sizes were one to six nucleotides, and the minimum repeat units were defined as 10, 5, 5, 4, 3 and 3 for mono-, di-, tri-, tetra-, penta- and hexa-nucleotides, respectively.

### Phylogenetic analysis

In addition to fourteen plastomes newly sequenced (thirteen *A*. subg. *Rhizirideum* plastomes and *A. condensatum* from subgenus Cepa), another fifty-three species were also selected (including thirty-six *Allium* species from our team, thirteen *Allium* species and four Amaryllidaceae outgroups downloaded from NCBI) (Additional file 4: Table S3) to infer the phylogenetic relationships. Seventy-seven CDSs were extracted from sixty-seven taxa by using Phylosuite and were multiple-aligned with MAFFT. The alignments were trimmed with trimAl and then concatenated in series for the phylogenetic analysis with Phylosuite to form a CDS dataset. Sixty-seven plastome sequences were multiple-aligned with MAFFT and trimmed with trimAl, leaving LSC, SSC and only one IR region to establish a cp dataset. The CDS and cp datasets were used to perform phylogenetic inferences, respectively. The Maximum Likelihood (ML) analysis was performed by RAxML v8.2.8 [112] with the GTR + G model and 1000 bootstrap replicates. The Bayes Inference (BI) analysis was performed by MrBayes v3.2.7 [113] with the substitution model GTR + I + Γ. The Markov chain Monte Carlo (MCMC) algorithm was run for one million generations, and one tree was sampled every 1000 generations. We then determined the MCMC convergence according to the average standard deviation of split frequencies (ASDSF) below 0.01. The first 20% of the trees were discarded as burn-in, and the remaining trees were used to generate consensus trees. Finally, online software Interactive Tree of Life (iTOL) was used to edit the phylogenetic trees [114].

### Selective pressure analysis

Thirteen studied species were used to calculate pairwise Ka/Ks ratios with KaKs Calculator v2.0 [115], and the average values were calculated to represent the Ka/Ks ratio of each gene. Seventy-seven CDSs of thirty-seven taxa were extracted and aligned with the software MUSCLE v5 [116] aligned by codons. The positive selection analyses, measured by the ratio (ω) of the non-synonymous substitution rate (Ka) to the synonymous substitution rate (Ks), were performed using the branch-site model in EasyCodeML v1.4 [117] and our subgenus lineage was designated. Positive, neutral, and purifying selection are demonstrated when the ratio ω > 1, w = 1, and ω < 1, respectively [118]. The log-likelihood values were tested (LRT) in accordance with [119]. The BEB method [120] was applied to compute the posterior probabilities of amino acid sites, and those with a higher posterior probability were determined to be under positive selection.

## Conclusions

Our work revealed that (1) the *Rhizirideum* plastomes have similar structures, (2) the phylogenetic position of the *Rhizirideum* species *A. polyrhizum* and *A. siphonanthum* should be reconsidered, (3) the plastome gene *ycf2* is under positive selection, probably contributing to the adaptability to the environment. Much remains to be investigated on the phylogenetic relationships of species in subgenus *Rhizirideum*, notably improving the sampling of *Allium* species.

## Supplementary Information

**Additional file 1: Fig S1.** Bulb shapes of 9 species. (A), *A.bidentatum*; (B), *A. mongolicum*; (C), *A. anisopodium*; (D), *A. tenuissimum*; (E), *A. senescens*; (F), *A. eduardii*; (G), *A. przewalskianum*; (H), *A. polyrhizum*; (I), *A. caespitosum.*

**Additional file 2: Table S1.** Current species in subgenus *Rhizirideum* (Bold fonts show the thirteen species selected in our study).

**Additional file 3: Table S2.** List of common CDSs in thirteen *Rhizirideum* plastomes for phylogenetic reconstrucion.

**Additional file 4:** **Table S3.** List of species and their accession numbers in GenBank included in the phylogenetic analysis (species in bold are from our team).

**Additional file 5:** **Table S4.** Collection locality and voucher information of twelve sequenced plastomes.

**Additional file 6: Table S5** **&** **S6 Table S4.** Codon usage of protein-coding genes of the thirteen *Rhizirideum* plastomes. Table S5. RSCU values of protein-coding genes of the thirteen *Rhizirideum* plastomes.

**Additional file 7: Table S7.** The repeat sequence distribution in the thirteen *Rhizirideum* plastomes.

**Additional file 8: Table S8.** Simple sequence repeats (SSRs) distribution in the thirteen *Rhizirideum* plastomes.

**Additional file 9:** **Table S9.** Results of selective pressure analysis in EasycodeMl with the branch-site model.

## Data Availability

All data generated or analysed during this study are included in this published article and its supplementary information files. The datasets analyzed during the current study are available in the NCBI GenBank repository (See supplementary Additional file 4: Table S3 for accessions).

## References

[1] Herden T, Hanelt P, Friesen N (2016). Phylogeny of Allium L. subgenus Anguinum (G. Don. Ex W.D.J. Koch) N. Friesen (Amaryllidaceae). Mol. Phylogenetics Evol.

[2] Linnaeus C: Species Plantarum: Exhibentes Plantas Rite Cognitas Ad Genera Relatas, Cum Differentiis Specificis, Nominibus Trivialibus, Synonymis Selectis, Locis Natalibus, Secundum Systema Sexuale Digestas; 1753.

[3] Regel E (1887). Allii species Asiae Centralis in Asia Media a Turcomania desertisque Araliensibus et Caspicis usque ad Mongolian crescentes. Acta Hort Petropol.

[4] Regel E (1875). Alliorum adhuc cognitorum monographia. Acta Hort. Petropol..

[5] Traub HP. The subgenera, sections and subsections of Allium L. Plant Life. 1968;24.

[6] Wendelbo P: New subgenera, sections and species of Allium. bot. notiser 1969, 122.

[7] Kamelin PB (1973). Florogeneticheskij analiz estestvennoj flory gornoj Srednej Azii.

[8] Friesen N, Fritsch RM, Blattner FR (2006). Phylogeny and new Intrageneric classification of Allium (Alliaceae) based on nuclear ribosomal DNA ITS sequences. Aliso.

[9] Li QQ, Zhou SD, He XJ, Yu Y, Zhang YC, Wei XQ (2010). Phylogeny and biogeography of Allium (Amaryllidaceae: Allieae) based on nuclear ribosomal internal transcribed spacer and chloroplast rps16 sequences, focusing on the inclusion of species endemic to China. Ann Bot.

[10] Friesen N, Smirnov S, Shmakov A, Herden T, Batlai O, Hurka H (2020). Allium species of section Rhizomatosa, early members of the central Asian steppe vegetation. Flora.

[11] Jang JE, Park JS, Jung JY, Kim DK, Yang S, Choi HJ (2021). Notes on Allium section Rhizirideum (Amaryllidaceae) in South Korea and northeastern China: with a new species from Ulleungdo Island. Phytokeys.

[12] Shopova M (1966). The nature and behaviour of supernumerary chromosomes in the Rhizirideum group of the genus Allium. Chromosoma.

[13] Friesen N. Systematics of the Siberian polyploid complex in subgenus Rhizirideum (Allium). In: Hanelt P, Hammer K, editors. The Genus Allium - Taxonomie Problemms and Genetic Resources. Proceedings of an International Symposium. Gatersleben. Germany; 1991, 1992. p. 55–66.

[14] Dubouzet J, Shinoda K, Murata N (1997). Phylogeny of Allium L. subgenus Rhizirideum (G. Don ex Koch) Wendelbo according to dot blot hybridization with randomly amplified DNA probes. Theor. Appl.

[15] Do GS, Seo BB (2000). Phyiogenetic relationships among Allium subg. Rhizirideum species based on the molecular variation of 5S rRNA genes. KJBS.

[16] Raamsdonk L, Ginkel VV, Kik C (2000). Phylogeny reconstruction and hybrid analysis in Allium subgenus Rhizirideum. Theor. Appl..

[17] Kim HH, Kang HW, Park YJ, Baek HJ, Gwag JK (2001). Phylogenic relationship of Allium species in subgenus Rhizirideum by PCR DNA fingerprint. Korean J Crop Sci.

[18] Lee NS (2001). Phylogenetic analyses of nuclear rDNA ITS sequences of Korean Allium L. subgenus Rhizirideum (Alliaceae). KJBS.

[19] Raamsdonk LV, Ensink W, Heusden A, Ginkel M, Kik C (2003). Biodiversity assessment based on cpDNA and crossability analysis in selected species of Allium subgenus Rhizirideum. Theor Appl.

[20] Ricroch A, Yockteng R, Brown S, Nadot S (2005). Evolution of genome size across some cultivated Allium species. Genome.

[21] Özler H, Pehlivan S. Pollen morphology of some Allium L. (Lilliaceae) taxa in turkey. Bangladesh J. Bot. 2010;39.

[22] Rola K. Cell Pattern and Ultrasculpture of Bulb Tunics of Selected Allium Species (Amaryllidaceae), and their Diagnostic Value. Acta Biol Crac Ser Bot. 2014.

[23] Li QQ, Zhou SD, Huang DQ, He XJ, Wei XQ (2016). Molecular phylogeny, divergence time estimates and historical biogeography within one of the world's largest monocot genera. Aob Plants.

[24] Sinitsyna T, Herden T, Friesen N. Dated phylogeny and biogeography of the Eurasian Allium section Rhizirideum (Amaryllidaceae). Plant Syst Evol. 2016;302.

[25] Li L, Hu Y, He M, Zhang B, Wu W, Cai P, et al. Comparative chloroplast genomes: insights into the evolution of the chloroplast genome of Camellia sinensis and the phylogeny of Camellia. BMC Genom. 2021;22(1). 10.1186/s12864-021-07427-2 PMC7912895 33637038

[26] Tang D, Wei F, Zhou R. Comparative analysis of chloroplast genomes of Kenaf cytoplasmic male sterile line and its maintainer line. Sci. Rep. 2021;11(1). 10.1038/s41598-021-84567-1 PMC7935921 33674697

[27] Wen F, Wu X, Li T, Jia M, Liu X, Liao L. The complete chloroplast genome of Stauntonia chinensis and compared analysis revealed adaptive evolution of subfamily Lardizabaloideae species in China. BMC Genom. 2021;22(1). 10.1186/s12864-021-07484-7 PMC7937279 33676415

[28] Yang X, Xie DF, Chen JP, Zhou SD, He XJ (2020). Comparative analysis of the complete chloroplast genomes in Allium subgenus Cyathophora (Amaryllidaceae): phylogenetic relationship and adaptive evolution. Biomed Res Int.

[29] Zyab C, Tao DA, Sv A, Fk B, Dmab C, Kt B, Hang SA (2021). Phylogenomics of Allium section Cepa (Amaryllidaceae) provides new insights on domestication of onion - ScienceDirect. Plant Divers.

[30] Xie DF, Huan-Xi YU, Price M, Xie C, He XJ. Phylogeny of Chinese Allium species in section Daghestanica and adaptive evolution of Allium (Amaryllidaceae, Allioideae) species revealed by the chloroplast complete genome. Front Plant Sci. 2019;10. 10.3389/fpls.2019.00460 PMC6503222 31114591

[31] Xie D, Tan J, Yu Y, Gui L, Su D, Zhou S, He X (2020). Insights into phylogeny, age and evolution of Allium (Amaryllidaceae) based on the whole plastome sequences. Ann Bot-London.

[32] Palmer JD, Osorio B, Aldrich J, Thompson WF (1987). Chloroplast DNA evolution among legumes: loss of a large inverted repeat occurred prior to other sequence rearrangements. Curr Genet.

[33] Tsudzuki J, Nakashima K, Tsudzuki T, Hiratsuka J, Shibata M, Wakasugi T, Sugiura M (1992). Chloroplast DNA of black pine retains a residual inverted repeat lacking rRNA genes: nucleotide sequences of trnQ, trnK, psbA, trnI and trnH and the absence of rps16. Mol Gen Genet.

[34] Lee HL, Jansen RK, Chumley TW, Kim KJ (2007). Gene relocations within chloroplast genomes of Jasminum and Menodora (Oleaceae) are due to multiple overlapping inversions. Mol Biol Evol.

[35] Wicke S, Schneeweiss GM, De Pamphilis CW, Kai FM, Quandt D (2011). The evolution of the plastid chromosome in land plants: gene content, gene order, gene function. Plant Mol Biol.

[36] Park S, Jansen RK, Park S (2015). Complete plastome sequence of Thalictrum coreanum (Ranunculaceae) and transfer of the rpl32 gene to the nucleus in the ancestor of the subfamily Thalictroideae. BMC Plant Biol.

[37] Zhu A, Guo W, Gupta S, Fan W, Mower J (2016). Evolutionary dynamics of the plastid inverted repeat: the effects of expansion, contraction, and loss on substitution rates. New Phytol.

[38] Huang Y, Cho S, Haryono M, Kuo C (2017). Complete chloroplast genome sequence of common bermudagrass (Cynodon dactylon (L.) Pers.) and comparative analysis within the family Poaceae. PloS One.

[39] Zhai W, Duan X, Zhang R, Guo C, Li L, Xu G, Shan H, Kong H, Ren Y (2019). Chloroplast genomic data provide new and robust insights into the phylogeny and evolution of the Ranunculaceae. Mol Phylogenet Evol.

[40] Ren T, Li ZX, Xie DF, Gui LJ, Peng C, Wen J, He XJ (2020). Plastomes of eight Ligusticum species: characterization, genome evolution, and phylogenetic relationships. BMC Plant Biol.

[41] Jin GH, Chen SY, Ting-Shuang YI, Zhang SD (2014). Characterization of the complete chloroplast genome of apple (Malus × domestica, Rosaceae). Plant Divers and Res.

[42] Liu Q, Li X, Li M, Xu W, Heslop-Harrison JS. Comparative chloroplast genome analyses of Avena: Insights into evolutionary dynamics and phylogeny. BMC Plant Biol. 2020;20(1). 10.1186/s12870-020-02621-y PMC7466839 32878602

[43] Ravi V, Khurana JP, Tyagi AK, Khurana P (2006). The chloroplast genome of mulberry: complete nucleotide sequence, gene organization and comparative analysis. Tree Genet Genomes.

[44] McKain MR, McNeal JR, Kellar PR, Eguiarte LE, Pires JC, Leebens-Mack J (2016). Timing of rapid diversification and convergent origins of active pollination within Agavoideae (Asparagaceae). Am J Bot.

[45] Bent AF, Kunkel BN, Dahlbeck D, Brown KL, Schmidt R, Giraudat J, Leung J, Staskawicz BJ (1994). RPS2 of Arabidopsis thaliana: a leucine-rich repeat class of plant disease resistance genes. Science.

[46] Downie SR, Jansen RK (2015). A comparative analysis of whole plastid genomes from the Apiales: expansion and contraction of the inverted repeat, mitochondrial to plastid transfer of DNA, and identification of highly divergent noncoding regions. Syst Bot.

[47] Goulding SE, Wolfe KH, Olmstead RG, Morden CW (1996). Ebb and flow of the chloroplast inverted repeat. Mol Gen Genet MGG.

[48] Wang RJ, Cheng CL, Chang CC, Wu CL, Su TM, Chaw SM. Dynamics and evolution of the inverted repeat-large single copy junctions in the chloroplast genomes of monocots. BMC Evol Biol. 2008;8. 10.1186/1471-2148-8-36 PMC2275221 18237435

[49] Wu C, Chaw S (2015). Evolutionary stasis in cycad Plastomes and the first case of Plastome GC-biased gene conversion. Genome Biol Evol.

[50] Raubeson LA, Jansen RK (1992). A rare chloroplast-DNA structural mutation is shared by all conifers. Biochem Syst Ecol.

[51] Chumley TW, Palmer JD, Mower JP, Matthew FH, Calie PJ, Boore JL, Jansen RK (2006). The complete chloroplast genome sequence of Pelargonium × hortorum: organization and evolution of the largest and Most highly rearranged chloroplast genome of land plants. Mol Biol Evol.

[52] Guisinger MM, Kuehl JV, Boore JL, Jansen RK. Extreme reconfiguration of plastid genomes in the angiosperm family Geraniaceae: rearrangements, repeats, and codon usage. Mol Biol Evol. 2010. 10.1093/molbev/msq229 20805190

[53] Grewe F, Guo W, Gubbels E, Hansen A, Mower J. Complete plastid genomes from Ophioglossum californicum, Psilotum nudum, and Equisetum hyemale reveal an ancestral land plant genome structure and resolve the position of Equisetales among monilophytes. BMC Evol Biol. 2013;13. 10.1186/1471-2148-13-8 PMC3553075 23311954

[54] Sun YX, Moore MJ, Meng AP, Soltis PS, Soltis DE, Li JQ, Wang HC (2013). Complete plastid genome sequencing of Trochodendraceae reveals a significant expansion of the inverted repeat and suggests a Paleogene divergence between the two extant species. PLoS One.

[55] Guo W, Felix G, Amie CC, Fan W, Duan Z, Adams RP, Schwarzbach AE, Mower JP (2014). Predominant and substoichiometric isomers of the plastid genome coexist within Juniperus plants and have shifted multiple times during Cupressophyte evolution. Genome Biol Evol.

[56] Ishihama A (2000). Functional modulation of Escherichia Coli RNA polymerase. Annu Rev Microbiol.

[57] Niu DK, Yang YF (2011). Why eukaryotic cells use introns to enhance gene expression: splicing reduces transcription-associated mutagenesis by inhibiting topoisomerase I cutting activity. Biol Direct.

[58] Petersen K, Schottler MA, Karcher D, Thiele W, Bock R (2011). Elimination of a group II intron from a plastid gene causes a mutant phenotype. Nucleic Acids Res.

[59] Huotari T, Korpelainen H (2012). Complete chloroplast genome sequence of Elodea canadensis and comparative analyses with other monocot plastid genomes. Gene.

[60] Liu J, Qi ZC, Zhao YP, Fu CX, Jenny XQ (2012). Complete cpDNA genome sequence of Smilax China and phylogenetic placement of Liliales--influences of gene partitions and taxon sampling. Mol Phylogenet Evol.

[61] Peredo EL, King UM, Les DH (2013). The plastid genome of Najas flexilis: adaptation to submersed environments is accompanied by the complete loss of the NDH complex in an aquatic angiosperm. PLoS One.

[62] Xie DF, Yu Y, Wen J, Huang J, He XJ (2020). Phylogeny and highland adaptation of Chinese species in Allium section Daghestanica (Amaryllidaceae) revealed by transcriptome sequencing. Mol Phylogenet Evol.

[63] Ogata H, Audic S, Renesto-Audiffren P, Fournier PE, Barbe V, Samson D, Roux V, Cossart P, Weissenbach J, Claverie JM (2001). Mechanisms of evolution in rickettsia conorii and R. prowazekii. Science.

[64] Lane CE, van den Heuvel K, Kozera C, Curtis BA, Parsons BJ, Bowman S, Archibald JM (2007). Nucleomorph genome of Hemiselmis andersenii reveals complete intron loss and compaction as a driver of protein structure and function. Proc Natl Acad Sci U S A.

[65] Smith DR, Lee RW (2008). Mitochondrial genome of the colorless green alga Polytomella capuana: a linear molecule with an unprecedented GC content. Mol Biol Evol.

[66] Dybvig K, Voelker LRL (2003). MOLECULAR BIOLOGY OF MYCOPLASMAS. Annu Rev Microbiol.

[67] Manen JF, Cuénoud P, Martinez MDP (1998). Intralineage variation in the pattern of rbcL nucleotide substitution. Plant Sys Evol.

[68] Wang L, Xing H, Yuan Y, Wang X, Saeed M, Tao J, Feng W, Zhang G, Song X, Sun X (2018). Genome-wide analysis of codon usage bias in four sequenced cotton species. PLoS One.

[69] Ernst JF (1988). Codon usage and gene expression. Trends Biotechnol.

[70] Morton BR (1998). Selection on the codon bias of chloroplast and cyanelle genes in different plant and algal lineages. J Mol Evol.

[71] Duan H, Zhang Q, Wang C, Li F, Tian F, Lu Y, Hu Y, Yang H, Cui G Analysis of codon usage patterns of the chloroplast genome in L reveals a preference for AT-ending codons as a result of major selection constraints, PEERJ 2021, 9:e10787. 10.7717/peerj.10787 PMC7819120 33552742

[72] Li HT, Yi TS, Gao LM, Ma PF, Zhang T, Yang JB, Gitzendanner MA, Fritsch PW, Cai J, Luo Y (2019). Origin of angiosperms and the puzzle of the Jurassic gap. Nat Plants.

[73] Guisinger MM, Kuehl JV, Boore JL, Jansen RK (2011). Extreme reconfiguration of plastid genomes in the angiosperm family Geraniaceae: rearrangements, repeats, and codon usage. Mol Biol Evol.

[74] Cho M, Kim H, Son HS (2019). Codon usage patterns of LT-ag genes in polyomaviruses from different host species. Virol J.

[75] Powell W, Morgante M, Andre C, McNicol JW, Machray GC, Doyle JJ, Tingey SV, Rafalski JA (1995). Hypervariable microsatellites provide a general source of polymorphic DNA markers for the chloroplast genome. Curr Biol.

[76] Cavalier-Smith T (2002). Chloroplast evolution: secondary symbiogenesis and multiple losses. Curr Biol.

[77] Roullier C, Rossel G, Tay D, McKey D, Lebot V (2011). Combining chloroplast and nuclear microsatellites to investigate origin and dispersal of New World sweet potato landraces. Mol Ecol.

[78] Huang J, Chen R, Li X. Comparative Analysis of the Complete Chloroplast Genome of Four Known Ziziphus Species. Genes (Basel). 2017;8(12). 10.3390/genes8120340 PMC5748658 29186778

[79] Xie DF, Li MJ, Tan JB, Price M, Xiao QY, Zhou SD, Yu Y, He XJ (2017). Phylogeography and genetic effects of habitat fragmentation on endemic Urophysa (Ranunculaceae) in Yungui plateau and adjacent regions. PLoS One.

[80] Tuler AC, Carrijo TT, Noia LR, Ferreira A, Peixoto AL, Da SFM (2015). SSR markers: a tool for species identification in Psidium (Myrtaceae). Mol Biol Rep.

[81] Jiang Y, Xu S, Wang R, Zhou J, Dou J, Yin Q, Wang R (2020). Characterization, validation, and cross-species transferability of EST-SSR markers developed from Lycoris aurea and their application in genetic evaluation of Lycoris species. BMC Plant Biol.

[82] Kapoor M, Mawal P, Sharma V, Gupta RC (2020). Analysis of genetic diversity and population structure in Asparagus species using SSR markers. J Genet Eng Biotechnol.

[83] Ogihara Y, Terachi T, Sasakuma T (1988). Intramolecular recombination of chloroplast genome mediated by short direct-repeat sequences in wheat species. Proc Natl Acad Sci U S A.

[84] Timme RE, Kuehl JV, Boore JL, Jansen RK (2007). A comparative analysis of the Lactuca and Helianthus (Asteraceae) plastid genomes: identification of divergent regions and categorization of shared repeats. Am J Bot.

[85] Weng ML, Blazier JC, Govindu M, Jansen RK (2014). Reconstruction of the ancestral plastid genome in Geraniaceae reveals a correlation between genome rearrangements, repeats, and nucleotide substitution rates. Mol Biol Evol.

[86] Yang Y, Zhou T, Duan D, Yang J, Feng L, Zhao G (2016). Comparative analysis of the complete chloroplast genomes of five Quercus species. Front Plant Sci.

[87] Zhang X, Zhou T, Kanwal N, Zhao Y, Bai G, Zhao G (2017). Completion of Eight Gynostemma BL. (Cucurbitaceae) Chloroplast Genomes: Characterization, Comparative Analysis, and Phylogenetic Relationships. Front Plant Sci.

[88] Xie F, Xie D, Xie C, Yu Y, Zhou S, He X, Subramanian S (2020). Adaptation evolution and phylogenetic analyses of species in Chinese Allium section Pallasia and related species based on complete chloroplast genome sequences. Biomed Res Int.

[89] Huang DQ, Yang JT, Zhou CJ, Zhou SD, He XJ (2014). Phylogenetic reappraisal of Allium subgenus Cyathophora (Amaryllidaceae) and related taxa, with a proposal of two new sections. J Med Plant Res.

[90] Iida S, Miyagi A, Aoki S, Ito M, Kadono Y, Kosuge K (2009). Molecular adaptation of rbcL in the heterophyllous aquatic plant Potamogeton. PLoS One.

[91] Wu Z, Liao R, Yang T, Dong X, Lan D, Qin R, Liu H (2020). Analysis of six chloroplast genomes provides insight into the evolution of Chrysosplenium (Saxifragaceae). BMC Genomics.

[92] Han Y, Liu X, Nan F, Feng J, Lv J, Liu Q, Xie S (2021). Analysis of adaptive evolution and coevolution of rbcL gene in the genus Galdieria (Rhodophyta). J Eukaryot Microbiol.

[93] Zhu B, Qian F, Hou Y, Yang W, Cai M, Wu X (2021). Complete chloroplast genome features and phylogenetic analysis of Eruca sativa (Brassicaceae). PLoS One.

[94] Shen J, Li X, Chen X, Huang X, Jin S. The Complete Chloroplast Genome of Carya cathayensis and Phylogenetic Analysis. Genes (Basel). 2022;13(2). 10.3390/genes13020369 PMC8871582 35205413

[95] Bathellier C, Yu L, Farquhar GD, Coote ML, Lorimer GH, Tcherkez G (2020). Ribulose 1,5-bisphosphate carboxylase/oxygenase activates O2 by electron transfer. Proc Natl Acad Sci.

[96] Boudreau E, Turmel M, Goldschmidt-Clermont M, Rochaix JD, Sivan S, Michaels A, Leu S (1997). A large open reading frame (orf1995) in the chloroplast DNA of Chlamydomonas reinhardtii encodes an essential protein. Mol Gen Genet.

[97] Drescher A, Ruf S, Calsa TJ, Carrer H, Bock R (2000). The two largest chloroplast genome-encoded open reading frames of higher plants are essential genes. Plant J.

[98] De Las RJ, Lozano JJ, Ortiz AR (2002). Comparative analysis of chloroplast genomes: functional annotation, genome-based phylogeny, and deduced evolutionary patterns. Genome Res.

[99] Nakai M (2015). YCF1: a green TIC: response to the de Vries et al commentary. The Plant Cell.

[100] Kikuchi S, Asakura Y, Imai M, Nakahira Y, Kotani Y, Hashiguchi Y, Nakai Y, Takafuji K, Bédard J, Hirabayashi-Ishioka Y (2018). A Ycf2-FtsHi Heteromeric AAA-ATPase complex is required for chloroplast protein import. Plant Cell.

[101] Yao B, Deng J, Liu J (2011). Variations between diploids and tetraploids of Allium przewalskianum, an important vegetable and/or condiment in the Himalayas. Chem Biodivers.

[102] Dierckxsens N, Mardulyn P, Smits G (2016). NOVOPlasty: De novo assembly of organelle genomes from whole genome data. Nucleic Acids Res.

[103] Qu XJ, Moore MJ, Li DZ, Yi TS (2019). PGA: a software package for rapid, accurate, and flexible batch annotation of plastomes. Plant Methods.

[104] Greiner S, Lehwark P, Bock R (2019). OrganellarGenomeDRAW (OGDRAW) version 1.3.1: expanded toolkit for the graphical visualization of organellar genomes. Nucleic Acids Res.

[105] Petkau A, Stuart-Edwards M, Stothard P, Domselaar GV, Valencia A (2010). Interactive microbial genome visualization with GView. Bioinformatics.

[106] Zhang D, Gao F, Jakovlic I, Zou H, Zhang J, Li WX, Wang GT (2020). PhyloSuite: an integrated and scalable desktop platform for streamlined molecular sequence data management and evolutionary phylogenetics studies. Mol Ecol Resour.

[107] Katoh K, Standley DM (2014). MAFFT: iterative refinement and additional methods. Methods Mol Biol.

[108] Capella-Gutierrez S, Silla-Martinez JM (2009). Gabaldon T: trimAl: a tool for automated alignment trimming in large-scale phylogenetic analyses. Bioinformatics.

[109] Frazer KA, Lior P, Alexander P, Rubin EM, Inna D. VISTA: computational tools for comparative genomics. Nucleic Acids Res. 2004:W273–9. 10.1093/nar/gkh458 PMC441596 15215394

[110] Rozas J, Ferrer-Mata A, Sánchez-DelBarrio JC, Guirao-Rico S, Librado P, Ramos-Onsins SE, Sánchez-Gracia A (2017). DnaSP 6: DNA sequence polymorphism analysis of large datasets. Mol Biol Evol.

[111] Stefan K, Choudhuri JV, Enno O, Chris S, Jens S, Robert G (2001). REPuter: the manifold applications of repeat analysis on a genomic scale. Nucleic Acids Res.

[112] Stamatakis A. RAxML version 8: a tool for phylogenetic analysis and post-analysis of large phylogenies. Bioinformatics. 2014;(9):30. 10.1093/bioinformatics/btu033 PMC3998144 24451623

[113] Ronquist F, Teslenko M, van der Mark P, Ayres D, Darling A, Ohna SH, Larget B, Liu L, Suchard MA, Huelsenbeck JP (2012). MrBayes 3.2: efficient Bayesian phylogenetic inference and model choice across a large model space. Sys. Biol.

[114] Letunic I, Bork P (2021). Interactive tree of life (iTOL) v5: an online tool for phylogenetic tree display and annotation. Nucleic Acids Res.

[115] Wang D, Zhang Y, Zhang Z, Zhu J, Yu J (2010). KaKs_Calculator 2.0: a toolkit incorporating gamma-series methods and sliding window strategies. Gen Proteo Bioinform.

[116] Edgar R: MUSCLE v5 enables improved estimates of phylogenetic tree confidence by ensemble bootstrapping. BioRxiv 2021(2021.06.20.449169).

[117] Gao F, Chen C, Arab DA, Du Z, He Y, Ho SYW. EasyCodeML: a visual tool for analysis of selection using CodeML. Ecol Evol. 2019:3891–8. 10.1002/ece3.5015 PMC6467853 31015974

[118] Yang Z, Nielson R (2002). Codon-substitution models for detecting molecular adaptation at individual sites along specific lineages. Mol Biol Evol.

[119] Lan Y, Sun J, Tian R, Bartlett DH, Li R, Wong YH (2017). Molecular adaptation in the world's deepest-living animal: insights from transcriptome sequencing of the hadal amphipod Hirondellea gigas. Mol Ecol.

[120] Yang Z, Wong WSW, Nielson R (2005). Bayes empirical Bayes inference of amino acid sites under positive selection. Mol Biol Evol.

