# Supplementary material for: Phylogeny and adaptive evolution of subgenus Rhizirideum (Amaryllidaceae, Allium) based on plastid genomes
Source: BMC Plant Biol. 2023 Feb 1;23:70. doi: 10.1186/s12870-022-03993-z (PMC9890777; doi:10.1186/s12870-022-03993-z)
Supplement: Supplementary file 1 — Additional file 1: Fig S1. Bulb shapes of 9 species. (A), A.bidentatum; (B), A. mongolicum; (C), A. anisopodium; (D), A. tenuissimum; (E), A. senescens; (F), A. eduardii; (G), A. przewalskianum; (H), A. polyrhizum; (I), A. caespitosum. [file 12870_2022_3993_MOESM1_ESM.pdf]

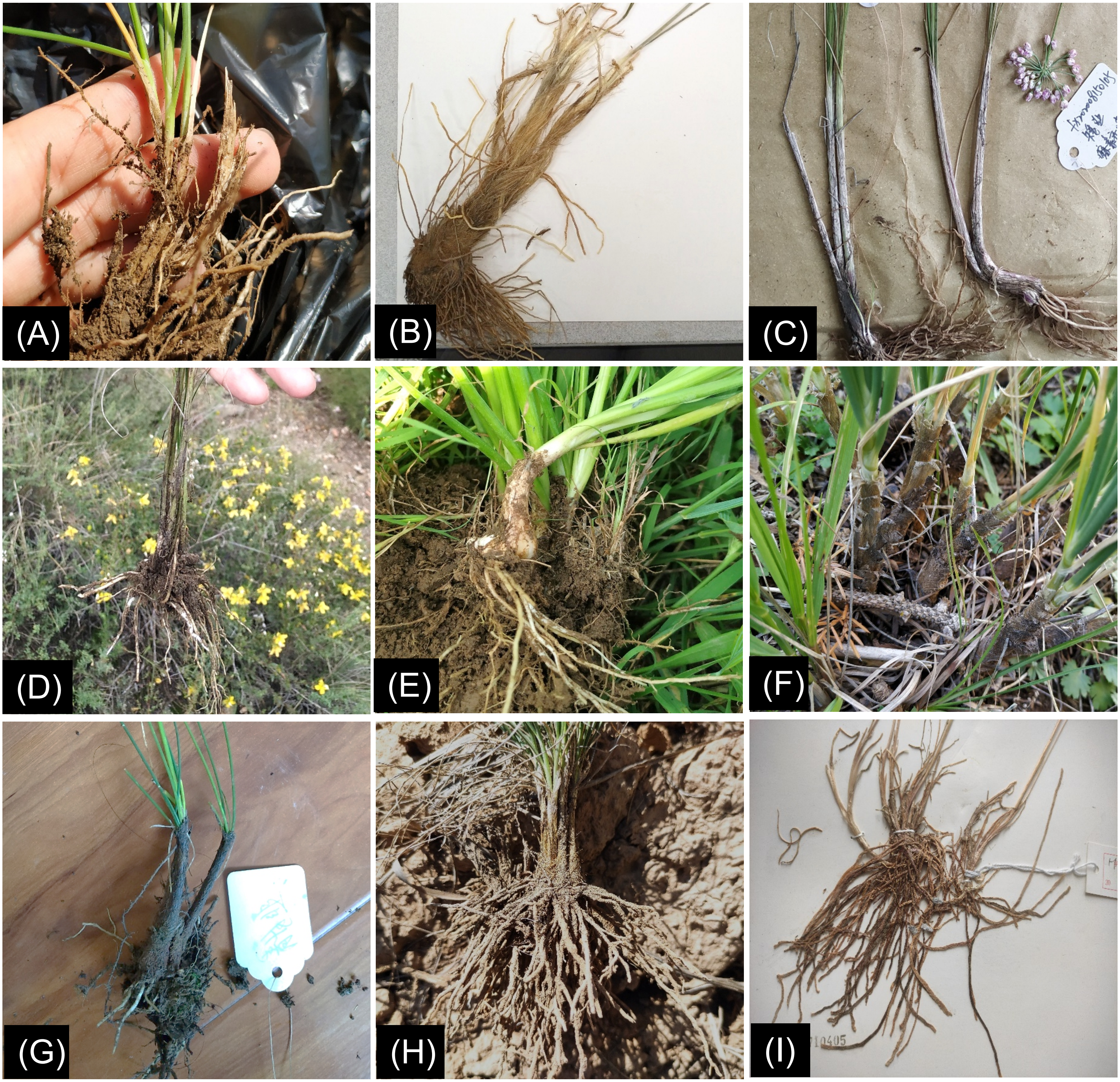

Figure S1. Bulb shapes of nine species. (A), *A.bidentatum*; (B), *A. mongolicum*; (C), *A. anisopodium*; (D), *A. tenuissimum*; (E), *A. senescens*; (F), *A. eduardii*; (G), *A. przewalskianum*; (H), *A. polyrhizum*; (I), *A. caespitosum*
